# xgt: a command-line interface for the Genome Taxonomy Database with cross-release taxonomic comparison

**DOI:** 10.1093/gigascience/giag086

**Published:** 2026-08-14

**Authors:** Anicet E T Ebou, Dominique K Koua, Adolphe Zézé

**Affiliations:** Laboratoire de Microbiologie, Biotechnologie et Bioinformatique, Institut National Polytechnique National Félix Houphouët-Boigny, BP 1093 Yamoussoukro, Côte d’Ivoire; Laboratoire de Microbiologie, Biotechnologie et Bioinformatique, Institut National Polytechnique National Félix Houphouët-Boigny, BP 1093 Yamoussoukro, Côte d’Ivoire; Laboratoire de Microbiologie, Biotechnologie et Bioinformatique, Institut National Polytechnique National Félix Houphouët-Boigny, BP 1093 Yamoussoukro, Côte d’Ivoire

## Background

The Genome Taxonomy Database (GTDB) provides a standardized, phylogenetically informed classification of bacterial and archaeal genomes [1], and has become the reference taxonomy in microbial ecology, comparative genomics, and environmental sequencing [2, 3]. GTDB releases are versioned (e.g., R214, R220, R232), and taxonomic assignments can change substantially between releases as new genomes are incorporated and phylogenetic placements are revised, directly affecting the reproducibility of studies conducted across release periods.

Despite exposing a REST API, GTDB provides no dedicated command-line interface. Researchers querying multiple accessions, performing batch searches, or tracking taxonomic changes across releases must write custom scripts or rely on general-purpose tools such as curl. Such tools require manual JSON parsing, provide no pagination, and offer no retry logic. GTDB-Tk [4] assigns taxonomy to new assemblies using a local reference tree but does not query the REST API. To our knowledge, no published tool provides a dedicated command-line interface for programmatic GTDB access covering batch retrieval, release-specific queries, and cross-release taxonomic comparison.

Here, we present xgt, an open-source command-line tool written in Rust [5], a systems programming language increasingly adopted for high-performance bioinformatics tools [6], that provides efficient, reproducible access to the GTDB REST API through 4 subcommands: search, genome, taxon, and diff. xgt is compiled as a self-contained binary with no runtime dependencies, available for Linux, macOS, and Windows at https://github.com/Ebedthan/xgt.

## Results

**Figure 1:**
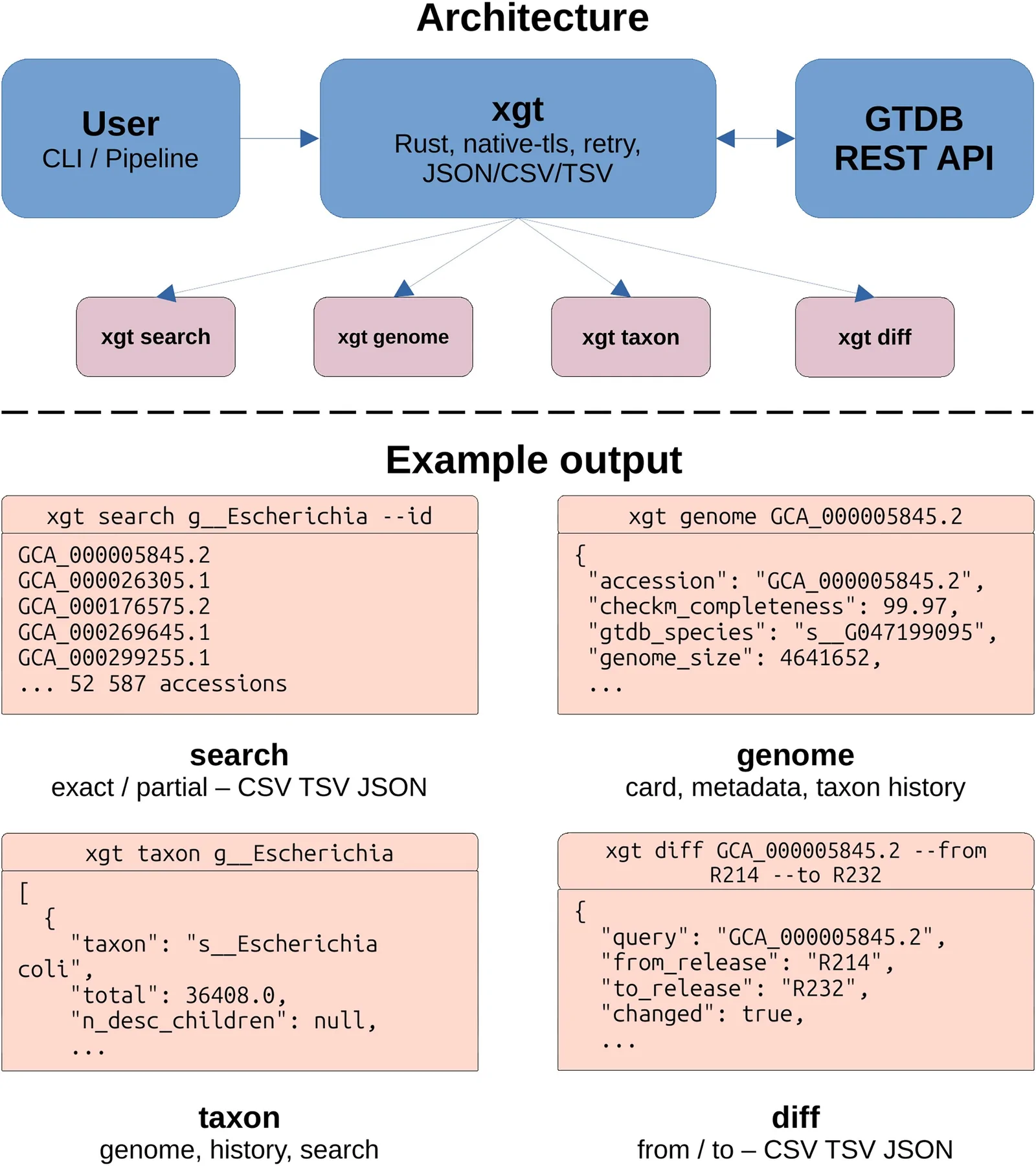
Overview of xgt architecture, subcommands, and representative outputs. The upper panel shows the 3-component architecture: the user interacts with xgt via the command-line or a pipeline; xgt communicates bidirectionally with the GTDB REST API using a blocking HTTP client with native TLS, automatic retry, and support for JSON, CSV, and TSV output. Four subcommands are exposed: search, genome, taxon, and diff. The lower panels show representative outputs for each subcommand. Output fields are truncated for display.

xgt exposes 4 subcommands through a unified interface (Fig. 1):

xgt <subcommand> [OPTIONS] [QUERY|ACCESSION|NAME]

All subcommands accept a single query as a positional argument, a file via –file, or standard input via –file -, enabling direct use in Unix pipelines. Output is controlled by –outfmt csv|tsv|json and can be directed to a single file (–out) or split into 1 file per query (–split).

A progress bar is displayed on standard error during batch runs, preserving stdout for downstream tools.

xgt search queries GTDB for genomes by name, accession, or taxonomy string with exact or partial matching, and returns results as CSV, TSV, or JSON. Pagination is transparent: xgt fetches all result pages sequentially, ensuring that queries that return large result sets (e.g., a search matching 52,000+ records) return complete results without truncation.

xgt genome retrieves full genome cards (nucleotide statistics, CheckM/CheckM2 quality metrics, NCBI metadata, GTDB classification), lightweight metadata, or complete taxonomic histories via mutually exclusive flags (–metadata, –history). The –release flag targets a specific GTDB release for card and metadata queries.

xgt taxon retrieves taxonomic records for named GTDB taxa, lists associated genome accessions (–genomes), or searches for taxon names in the current or all historical GTDB releases (–search, –all).

xgt diff addresses the principal gap in existing GTDB tooling. To our knowledge, no previously published tool provides a dedicated command-line interface to compute per-rank taxonomic changes between arbitrary GTDB release pairs directly via the live API without requiring a local database installation. xgt diff queries the existing /genome/{accession}/taxon-history endpoint and compares the 7-rank GTDB classification at a source release (–from) and a target release (–to; defaults to the most recent available). For each accession, the output reports a changed flag, per-rank change records carrying the old and new assignment, and full taxonomy snapshots at both releases. For example, comparing GCA_000005845.2 between R214 and R232 reveals a species-level reclassification from s__Escherichia coli to s__G047199095 sp047199095.

## Performance

We benchmarked xgt against curl + jq and python + requests on the search –id task across 3 genus sizes using hyperfine v1.20.0 [7] (10 timed runs, 3 warmup runs; full protocol in Supplementary Methods). xgt was consistently fastest (Table S1). For the medium query (g__Bacillus, 14 API pages), the median completion time was 1.50 versus 6.41 s for curl + jq (4.27x faster), reflecting per-page process invocation overhead. For the large query (g__Escherichia, 53 pages), network jitter dominated; the median times of 5.3, 13.1, and 25.2 s for xgt, python, and curl + jq, respectively, should be interpreted as real-world workflow efficiency rather than isolated computational benchmarks (Table S1).

## Biological validation

To demonstrate the biological utility of xgt diff, we retrieved the 46,827 R232 species-representative genomes assigned to p__Pseudomonadota using xgt search. Of these, 26,112 (55.8%) were absent from R214 and excluded from comparison, reflecting the substantial expansion of the p__Pseudomonadota genome set between the 2 releases, a more than 2-fold increase in representative diversity within this phylum alone. Of the remaining 20,715 genomes present in both releases, 7,941 (38.3%) were reclassified at 1 or more ranks (Table S2). Changes were concentrated at the species (19.7%) and genus (15.6%) levels, with higher ranks largely stable, consistent with the known dynamics of GTDB revision in which most updates reflect refined species circumscriptions and genus-level phylogenetic restructuring rather than changes to higher-rank framework taxa. A subset of 3,636 genomes (45.8% of reclassified) changed at 2 or more ranks simultaneously, indicating genuine phylogenetic repositioning.

These results illustrate that even between consecutive GTDB releases, a non-trivial proportion of reference genomes receive updated assignments that would silently affect taxonomic profiles, ecological comparisons, and database cross-references if release versions are not tracked explicitly. xgt diff provides a reproducible, single-command approach to this audit for any user-defined genome set.

## Discussion

xgt fills a practical gap in the GTDB ecosystem: to our knowledge, no existing tool provides a dedicated command-line interface for programmatic access to the GTDB REST API without requiring a local database installation, and xgt diff is, to our knowledge, the only tool that performs reproducible, scriptable cross-release taxonomic comparison operating directly on genome accessions via the live API. The biological validation demonstrates that reclassification between GTDB releases is not rare (38.3% of the 20,715 p__Pseudomonadota representatives present in both R214 and R232 changed between releases) with direct consequences for the reproducibility of amplicon, MAG, and reference database workflows.

The reclassification pattern observed in p__Pseudomonadota reflects the known dynamics of GTDB revision: most changes between releases arise from the integration of newly sequenced genomes that refine species boundaries and reshape genus-level circumscriptions, rather than from fundamental changes to higher-rank framework taxa. The concentration of changes at the species (19.7%) and genus (15.6%) levels is consistent with this, though reclassification at the family (14.1%) and order (10.3%) levels was also substantial, indicating that phylogenetic restructuring extended well beyond species circumscription. For downstream analyses, these changes have concrete consequences: species-level assignments from a published amplicon study may refer to different phylogenetic entities in a newer release; MAG catalogues built against R214 may map reads to genera that were synonymized or split in R232; and cross-study comparisons that do not report the GTDB release version are inherently ambiguous. xgt diff makes release-aware taxonomy a tractable step in routine workflows rather than a manual post-processing burden.

Several tools offer complementary functionality worth distinguishing from xgt. TaxonKit with gtdb-taxdump [8] can compute taxonomic changes between GTDB releases by diffing locally downloaded taxonomy dumps. This approach is well suited to bulk offline analysis but requires downloading and maintaining local database files of several gigabytes, and operates on taxon IDs rather than genome accessions. xgt diff operates directly against the live GTDB REST API without any local installation, accepts genome accessions as input, and returns complete taxonomy snapshots at both releases for contextual interpretation. The 2 approaches are complementary: TaxonKit is preferable for large-scale offline batch analysis, while xgt is suited to interactive use and integration into API-dependent pipelines. Workflow management systems such as Snakemake [9] and Nextflow [10] can orchestrate xgt as a step within larger genomic pipelines, and R packages such as taxize provide NCBI-based taxonomy utilities; none of these tools expose GTDB REST API functionality directly, and they are better understood as composable partners than as alternatives to xgt.

Current limitations include sequential pagination (throughput bounded by per-page API latency), API-dependent operation (no offline mode), and partial release filtering (the –release flag applies only to genome card and metadata endpoints; search and taxon endpoints always return the current release). Future development will introduce tolerant deserialization to handle API type changes without tool updates, and parallel pagination with rate-limit management. As GTDB continues to expand, from ∼317,000 genomes in R207 to over 901,000 in R232 [1], scalable, release-aware programmatic access will become increasingly important. xgt is designed to grow with the database and serve as a stable foundation for GTDB-dependent workflows.

## Availability of source code and requirements

- Project name: xgt
- Project home page: https://github.com/Ebedthan/xgt
- Operating system(s): Linux (x86_64), macOS (x86_64, Apple Silicon), Windows (x86_64)
- Programming language: Rust (edition 2021, MSRV 1.85)
- Other requirements: None (self-contained binary); Rust 1.85 or later required to build from source
- License: MIT and Apache 2.0 (dual licence)
- RRID: SCR_028473
- bio.tools ID: xgt

## Additional files

**Supplementary Table S1:** Wall-time benchmarks for the search –id task across 3 genus sizes. Values are mean, median, and standard deviation over 10 runs (3 warmup runs; GTDB release R232, residential broadband, Intel Core i7-8650 U). For *g*__*Escherichia* the coefficient of variation exceeds 100% due to network jitter over 53 API pages; the median is the more appropriate central tendency measure. Relative speedup is with respect to xgt (the fastest tool in each group).

**Supplementary Table S2:** Taxonomic reclassification rates between GTDB releases R214 and R232 for species-representative genomes assigned to *p__Pseudomonadota* (*n* = 20,715, genomes present in both R214 and R232 out of 46,827 R232 representatives). Percentages in the last column are of all 20,715 analysed genomes.

**Supplementary Table S3:** Comparison of xgt with related tools for GTDB data access. curl/wget are general-purpose HTTP tools that can query the API but provide no pagination, retry logic, structured parsing, or typed output. GTDB-Tk classifies novel genome assemblies against the GTDB reference phylogeny and does not query the REST API [3]. TaxonKit with gtdb-taxdump [4] can compute cross-release taxonomic changes from locally downloaded GTDB taxonomy dumps, but requires maintaining local database files and operates on taxon IDs rather than genome accessions. Many users also interact with GTDB programmatically via custom Python scripts or Jupyter notebooks; while these approaches offer flexibility, they require per-user implementation of pagination, error handling, and output formatting that xgt provides out of the box. ✓: supported; ×: not supported; N/A: not applicable to the tool’s primary function.

## List of abbreviations

API: Application Programming Interface; CLI: Command-Line Interface; CSV: Comma-Separated Values; GTDB: Genome Taxonomy Database; JSON: JavaScript Object Notation; MAG: Metagenome-Assembled Genome; REST: Representational State Transfer; TLS: Transport Layer Security; TSV: Tab-Separated Values.

## Supplementary Material

giag086_Supplemental_File

giag086_GIGA-D-26-00216_Original_Submission

giag086_GIGA-D-26-00216_Revision_1

giag086_GIGA-D-26-00216_Revision_2

giag086_Reviewer_1_Report_Original_Submission Reviewer 1 -- 7/9/2026

giag086_Reviewer_1_Report_Revision_1 Reviewer 1 -- 7/30/2026

giag086_Reviewer_2_Report_Original_Submission Reviewer 2 -- 7/21/2026

giag086_Reviewer_2_Report_Revision_1 Reviewer 2 -- 8/6/2026

## Data Availability

The datasets supporting the results of this article are available in the GitHub repository [11].
