# Supplementary material for: xgt: a command-line interface for the Genome Taxonomy Database with cross-release taxonomic comparison
Source: Gigascience. 2026 Aug 14;15:giag086. doi: 10.1093/gigascience/giag086 (PMC13505384; doi:10.1093/gigascience/giag086)
Supplement: Supplementary file 1 [file giag086_giga-d-26-00216_original_submission.pdf]

GigaScience

xgt: a command-line interface for the Genome Taxonomy Database with cross-release taxonomic comparison

--Manuscript Draft--

**Manuscript Number:**	GIGA-D-26-00216
**Full Title:**	xgt: a command-line interface for the Genome Taxonomy Database with cross-release taxonomic comparison
**Article Type:**	Brief Communication
**Funding Information:**	
**Abstract:**	Background:<br>The Genome Taxonomy Database (GTDB) provides a standardised, phylogenetically informed classification of bacterial and archaeal genomes, widely used as the reference taxonomy in microbial ecology and comparative genomics. Despite exposing a REST API, GTDB lacks a dedicated command-line interface, leaving researchers to write custom scripts that lack pagination for large result sets, retry logic for transient failures, and support for tracking taxonomic reclassifications across release versions.<br>Findings:<br>We present xgt, an open-source command-line tool written in Rust that provides efficient, flexible access to the GTDB REST API through four subcommands. xgt search queries GTDB for genomes by name, accession, or taxonomy with exact or partial matching. xgt genome retrieves full genome cards, lightweight metadata, or complete taxonomic histories. xgt taxon explores taxon lineages and associated genome sets. xgt diff computes per-rank taxonomic changes for a genome between any two GTDB releases, a capability not previously available in a dedicated GTDB command-line tool, to our knowledge. All subcommands support batch processing from files or standard input, transparent pagination for arbitrarily large result sets, structured output in JSON, CSV, and TSV formats, per-item file splitting for pipeline integration, and automatic retry with exponential backoff. xgt is compiled as a self-contained binary, with no external runtime dependencies, available for Linux, macOS, and Windows.<br>Conclusions:<br>xgt fills a practical gap in the GTDB ecosystem by providing a robust, pipeline-friendly interface that handles the operational realities of large-scale programmatic querying. xgt is freely available under the MIT and Apache~2.0 dual licence at https://github.com/Ebedthan/xgt.
**Corresponding Author:**	Anicet E. T. Ebou, Ph.D.<br>Institut National Polytechnique Felix Houphouet-Boigny<br>Yamoussoukro, CÔTE D'IVOIRE
**Corresponding Author Secondary Information:**	
**Corresponding Author's Institution:**	Institut National Polytechnique Felix Houphouet-Boigny
**Corresponding Author's Secondary Institution:**	
**First Author:**	Anicet E. T. Ebou, Ph.D.
**First Author Secondary Information:**	
**Order of Authors:**	Anicet E. T. Ebou, Ph.D.
	Dominique K. Koua, Ph.D.
	Adolphe Zézé, Ph.D.
**Order of Authors Secondary Information:**	
**Additional Information:**	
**Question**	**Response**

Powered by Editorial Manager® and ProduXion Manager® from Aries Systems Corporation

Are you submitting this manuscript to a special series or article collection?	No
**Experimental design and statistics**<br><br>Full details of the experimental design and statistical methods used should be given in the Methods section, as detailed in our Minimum Standards Reporting Checklist. Information essential to interpreting the data presented should be made available in the figure legends.<br><br>Have you included all the information requested in your manuscript?	Yes
**Resources**<br><br>A description of all resources used, including antibodies, cell lines, animals and software tools, with enough information to allow them to be uniquely identified, should be included in the Methods section. Authors are strongly encouraged to cite Research Resource Identifiers (RRIDs) for antibodies, model organisms and tools, where possible.<br><br>Have you included the information requested as detailed in our Minimum Standards Reporting Checklist?	Yes
**Availability of data and materials**<br><br>All datasets and code on which the conclusions of the paper rely must be either included in your submission or deposited in publicly available repositories (where available and ethically appropriate), referencing such data using a unique identifier in the references and in the "Availability of Data and Materials" section of your manuscript.<br><br>Have you have met the above requirement as detailed in our Minimum	Yes

Powered by Editorial Manager® and ProduXion Manager® from Aries Systems Corporation

Standards Reporting Checklist?	
GigaScience has policies and guidelines in place for the use of generative AI-writing tools such as ChatGPT. If you have used such writing tools to assist with writing the manuscript this must be declared and cited in the text. Authors should not list AI-writing tools and other AI-assisted technologies as an author or co-author and should acknowledge that they are fully responsible for text generated or refined by AI-writing tools.<p><br><br>A summary of use (particularly in the introduction or among methods) needs to be included at the end of the paper, and the outputs should also be included as a supplementary file hosted in GigaDB or other open repositories. Please read our guidelines for more information. <p><br><br>By submitting to GigaScience, you are aware of the journal's AI-writing tools policy, and if you have declared use of such tools below, you have acknowledged this where appropriate in your manuscript and have made a summary of use and outputs available. <p><br><b>AI-assisted writing tools have been used in the preparation of this manuscript?	No

Manuscript

Click here to access/download;Manuscript;brief_communication.pdf

GigaScience, 2026, 1–4

doi: xx.xxxx/xxxx
Manuscript in Preparation
Brief Communication

BRIEF COMMUNICATION

xgt: a command-line interface for the Genome Taxonomy Database with cross-release taxonomic comparison

Anicet E. T. Ebou[1,*], Dominique K. Koua[1] and Adolphe Zézé[1]

[1]Laboratoire de Microbiologie, Biotechnologie et Bioinformatique, Institut National Polytechnique Félix Houphouët-Boigny, BP 1093 Yamoussoukro, Côte d'Ivoire

[*]ediman.ebou@inphb.ci

Key words: GTDB; Microbial taxonomy; Genomic database; Taxonomic comparison; Command-line tool; Rust.

Background

The Genome Taxonomy Database (GTDB) provides a standardised, phylogenetically informed classification of bacterial and archaeal genomes [1], and has become the reference taxonomy in microbial ecology, comparative genomics, and environmental sequencing [2, 3]. GTDB releases are versioned (e.g. R214, R220, R232), and taxonomic assignments can change substantially between releases as new genomes are incorporated and phylogenetic placements are revised, directly affecting the reproducibility of studies conducted across release periods.

Despite exposing a REST API, GTDB provides no dedicated command-line interface. Researchers querying multiple accessions, performing batch searches, or tracking taxonomic changes across releases must write custom scripts or rely on general-purpose tools such as `curl`. Such tools requires manual JSON parsing, provide no pagination, and offer no retry logic. `GTDB-Tk` [4] assigns taxonomy to new assemblies using a local reference tree but does not query the REST API. To our knowledge, no published tool provides a dedicated command-line interface for programmatic GTDB access covering batch retrieval, release-specific queries, and cross-release taxonomic comparison.

Here we present `xgt`, an open-source command-line tool written in Rust [5] that provides efficient, reproducible access to the GTDB REST API through four subcommands: `search`, `genome`, `taxon`, and `diff`. `xgt` is compiled as a self-contained binary with no runtime dependencies, available for Linux, macOS, and Windows at https://github.com/Ebedthan/xgt.

Results

`xgt` exposes four subcommands through a unified interface (Figure 1):

```
xgt <subcommand> [OPTIONS]
↪ [QUERY|ACCESSION|NAME]
```

All subcommands accept a single query as a positional argument, a file via `--file`, or standard input via `--file -`, enabling direct use in Unix pipelines. Output is controlled by `--outfmt csv|tsv|json` and can be directed to a single file (`--out`) or split into one file per query (`--split`). A progress bar is displayed on standard error during batch runs, preserving stdout for downstream tools.

`xgt search` queries GTDB for genomes by name, accession, or taxonomy string with exact or partial matching, and returns results as CSV, TSV, or JSON. Pagination is transparent: `xgt` fetches all result pages sequentially, ensuring that queries on large genera (*g__Escherichia*, over 52 000 genomes) complete without truncation.

`xgt genome` retrieves full genome cards (nucleotide statistics, CheckM/CheckM2 quality metrics, NCBI metadata, GTDB classification), lightweight metadata, or complete taxonomic histories via mutually exclusive flags (`--metadata`, `--history`). The `--release` flag targets a specific GTDB release for card and metadata queries.

`xgt taxon` retrieves taxonomic records for named GTDB taxa, lists associated genome accessions (`--genomes`), or searches for taxon names in the current or all historical GTDB releases (`--search`, `--all`).

`xgt diff` addresses the principal gap in existing GTDB tooling. To our knowledge, no previously published tool computes per-rank taxonomic changes between arbitrary GTDB release pairs. `xgt diff` queries the existing `/genome/{accession}/taxon-history` endpoint

Compiled on: July 1, 2026.
Draft manuscript prepared by the author.

and compares the seven-rank GTDB classification at a source release (`--from`) and a target release (`--to`; defaults to the most recent available). For each accession, the output reports a `changed` flag, per-rank change records carrying the old and new assignment, and full taxonomy snapshots at both releases. For example, comparing `GCA_000005845.2` between R214 and R232 reveals a species-level reclassification from `s__Escherichia coli` to `s__G047199095 sp047199095`.

Performance

We benchmarked `xgt` against `curl + jq` and `python + requests` on the `search --id` task across three genus sizes using `hyperfine` 1.20.0 [6] (10 timed runs, 3 warmup runs; full protocol in Supplementary Methods). `xgt` was consistently fastest (Table S1). For the medium query (*g__Bacillus*, 14 API pages), the median completion time was 1.50 s versus 6.41 s for `curl + jq` (4.27× faster), reflecting per-page process invocation overhead. For the large query (*g__Escherichia*, 53 pages), network jitter dominated; the median times of 5.3, 13.1, and 25.2 s for `xgt`, `python`, and `curl + jq` respectively should be interpreted as real-world workflow efficiency rather than isolated computational benchmarks (Table S1).

Biological validation

To demonstrate the biological utility of `xgt diff`, we analysed taxonomic reclassification between GTDB releases R214 and R232 for all 20 715 species representative genomes assigned to *p__Pseudomonadota*. Of these, 7 941 (38.3%) were reclassified at one or more ranks (Table S2). Changes were concentrated at the species (19.7%) and genus (15.6%) levels, with higher ranks largely stable, consistent with the known dynamics of GTDB revision in which most updates reflect refined species circumscriptions and genus-level phylogenetic restructuring rather than changes to higher-rank framework taxa. A subset of 3 636 genomes (45.8% of reclassified) changed at two or more ranks simultaneously, indicating genuine phylogenetic repositioning. These results illustrate that even between consecutive GTDB releases, a non-trivial proportion of reference genomes receive updated assignments that would silently affect taxonomic profiles, ecological comparisons, and database cross-references if release versions are not tracked explicitly. `xgt diff` provides a reproducible, single-command approach to this audit for any user-defined genome set.

Discussion

`xgt` fills a practical gap in the GTDB ecosystem: it is the first dedicated command-line tool for programmatic access to the GTDB REST API, and `xgt diff` is, to our knowledge, the first tool to provide reproducible, scriptable cross-release taxonomic comparison for user-defined genome sets. The biological validation demonstrates that reclassification between GTDB releases is not rare (38.3% of *p__Pseudomonadota* representatives changed between R214 and R232) with direct consequences for the reproducibility of amplicon, MAG, and reference database workflows.

Current limitations include sequential pagination (throughput bounded by per-page API latency), API-dependent operation (no offline mode), and partial release filtering (the `--release` flag applies only to genome card and metadata endpoints; search and taxon endpoints always return the current release). Future development will introduce tolerant deserialisation to handle API type changes without tool updates, and parallel pagination with rate-limit management. As GTDB continues to expand, from approximately 258 000 genomes in R207 to over 400 000 in R232 [1], scalable, release-aware programmatic access will become increasingly important. `xgt` is designed to grow with the database and serve as a stable foundation for GTDB-dependent workflows.

Availability of supporting source code and requirements

- **Project name:** xgt
- **Project home page:** https://github.com/Ebedthan/xgt
- **Operating system(s):** Linux (x86_64), macOS (x86_64, Apple Silicon), Windows (x86_64)
- **Programming language:** Rust (edition 2021, MSRV 1.85)
- **Other requirements:** None (self-contained binary); Rust 1.85 or later required to build from source
- **License:** MIT and Apache 2.0 (dual licence)
- **RRID:** SCR_028473
- **bio.tools ID:** xgt

Benchmark and biological validation scripts: https://github.com/Ebedthan/xgt_article.

Abbreviations

API: Application Programming Interface; **CLI**: Command-Line Interface; **CSV**: Comma-Separated Values; **GTDB**: Genome Taxonomy Database; **JSON**: JavaScript Object Notation; **MAG**: Metagenome-Assembled Genome; **REST**: Representational State Transfer; **TLS**: Transport Layer Security; **TSV**: Tab-Separated Values.

Consent for publication

Not applicable

Competing Interests

The authors declare no competing interest.

Funding

Not applicable.

Author's Contributions

Anicet E. T. Ebou: Conceptualization, Methodology, Software, Writing - Original draft. **Dominique K. Koua**: Writing - Review & editing, Methodology, Conceptualization. **Adolphe Zézé**: Writing - Review & editing, Supervision.

Acknowledgements

Not applicable.

References

1. Parks DH, Chuvochina M, Rinke C, Mussig AJ, Chaumeil PA, Hugenholtz P. GTDB: An Ongoing Census of Bacterial and Archaeal Diversity through a Phylogenetically Consistent, Rank Normalized and Complete Genome-Based Taxonomy. Nucleic Acids Research 2022 Jan;50(D1):D785–D794.
2. Riesco R, Trujillo ME. Update on the Proposed Minimal Standards for the Use of Genome Data for the Taxonomy of Prokary-

otes. International Journal of Systematic and Evolutionary Microbiology 2024;74(3):006300.
3. Clagnan E, Costanzo M, Visca A, Di Gregorio L, Tabacchioni S, Colantoni E, et al. Culturomics- and Metagenomics-Based Insights into the Soil Microbiome Preservation and Application for Sustainable Agriculture. Frontiers in Microbiology 2024 Oct;15.
4. Chaumeil PA, Mussig AJ, Hugenholtz P, Parks DH. GTDB-Tk: A Toolkit to Classify Genomes with the Genome Taxonomy Database. Bioinformatics 2020 Mar;36(6):1925–1927.
5. Klabnik S, Nichols C. The Rust Programming Language. No Starch Press; 2018.
6. Peter D, hyperfine: A command-line benchmarking tool; 2023. https://github.com/sharkdp/hyperfine.

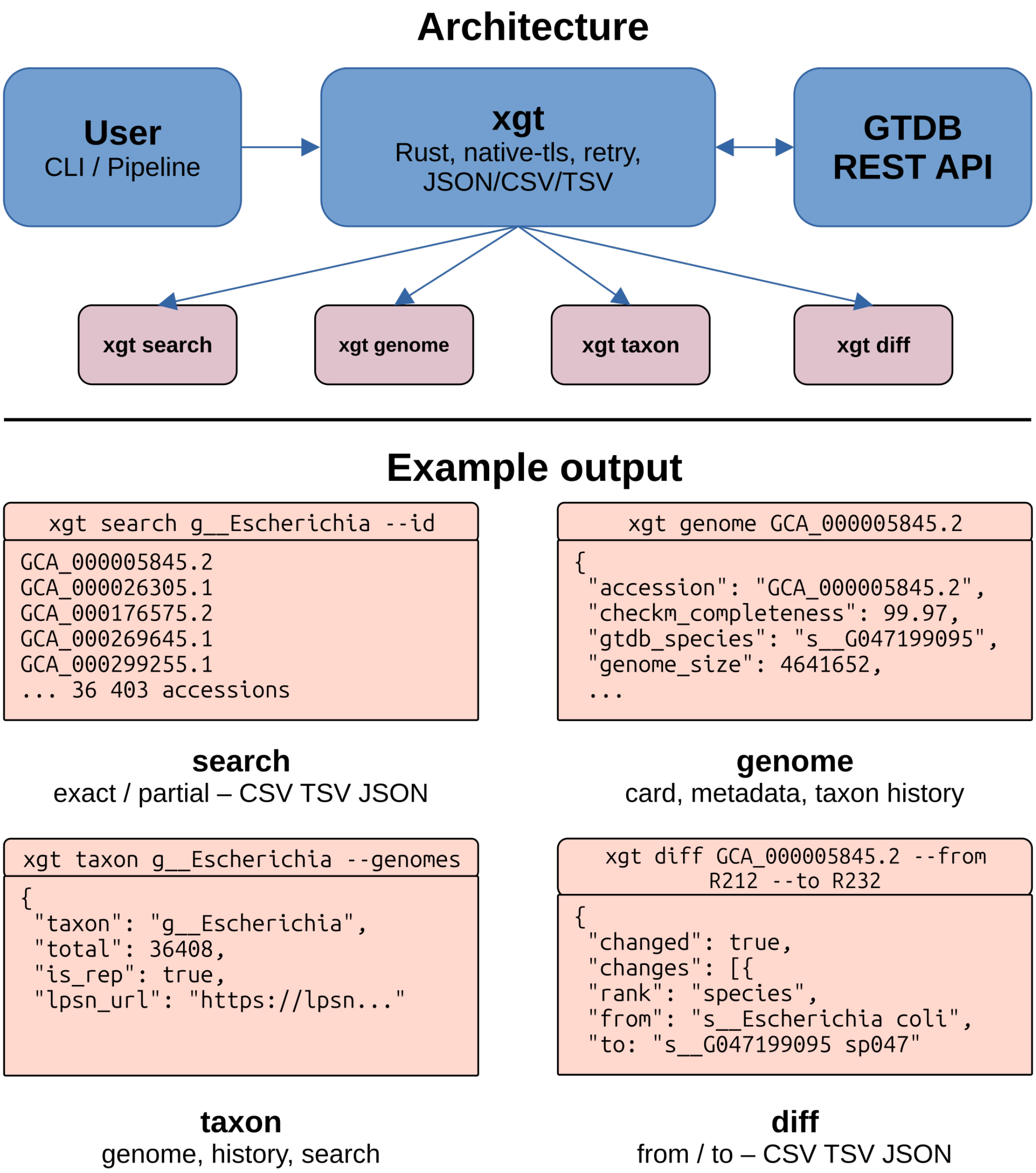

Figure 1. Overview of `xgt` architecture, subcommands, and representative outputs. The upper panel shows the three-component architecture: the user interacts with `xgt` via the command line or a pipeline; `xgt` communicates bidirectionally with the GTDB REST API using a blocking HTTP client with native TLS, automatic retry, and support for JSON, CSV, and TSV output. Four subcommands are exposed: `search`, `genome`, `taxon`, and `diff`. The lower panels show representative outputs for each subcommand. Output fields are truncated for display.

Figure Click here to access/download;Figure;fig1.png

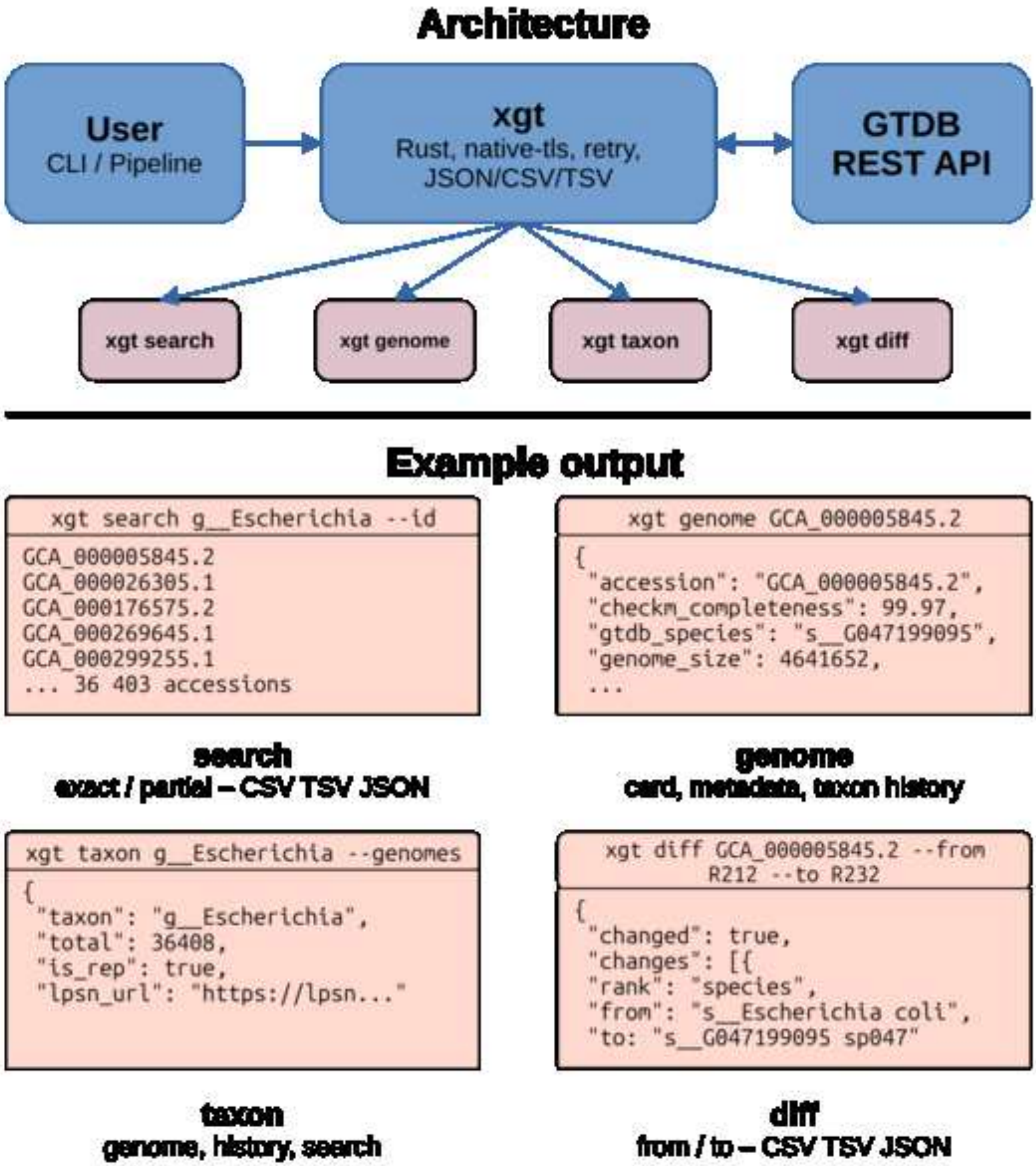

Manuscript with line and page number

Click here to access/download
Supplementary Material
xgt_article.docx

Click here to access/download
Supplementary Material
supplementary_file.pdf
