# Supplementary material for: xgt: a command-line interface for the Genome Taxonomy Database with cross-release taxonomic comparison
Source: Gigascience. 2026 Aug 14;15:giag086. doi: 10.1093/gigascience/giag086 (PMC13505384; doi:10.1093/gigascience/giag086)
Supplement: Supplementary file 2 [file giag086_giga-d-26-00216_revision_1.pdf]

GigaScience

xgt: a command-line interface for the Genome Taxonomy Database with cross-release taxonomic comparison

--Manuscript Draft--

**Manuscript Number:**	GIGA-D-26-00216R1
**Full Title:**	xgt: a command-line interface for the Genome Taxonomy Database with cross-release taxonomic comparison
**Article Type:**	Brief Communication
**Funding Information:**	
**Abstract:**	Background:<br>The Genome Taxonomy Database (GTDB) provides a standardised, phylogenetically informed classification of bacterial and archaeal genomes, widely used as the reference taxonomy in microbial ecology and comparative genomics. Despite exposing a REST API, GTDB lacks a dedicated command-line interface, leaving researchers to write custom scripts that lack pagination for large result sets, retry logic for transient failures, and support for tracking taxonomic reclassifications across release versions.<br>Findings:<br>We present xgt, an open-source command-line tool written in Rust that provides efficient, flexible access to the GTDB REST API through four subcommands. xgt search queries GTDB for genomes by name, accession, or taxonomy with exact or partial matching. xgt genome retrieves full genome cards, lightweight metadata, or complete taxonomic histories. xgt taxon explores taxon lineages and associated genome sets. xgt diff computes per-rank taxonomic changes for a genome between any two GTDB releases, a capability not previously available in a dedicated GTDB command-line tool, to our knowledge. All subcommands support batch processing from files or standard input, transparent pagination for arbitrarily large result sets, structured output in JSON, CSV, and TSV formats, per-item file splitting for pipeline integration, and automatic retry with exponential backoff. xgt is compiled as a self-contained binary, with no external runtime dependencies, available for Linux, macOS, and Windows.<br>Conclusions:<br>xgt fills a practical gap in the GTDB ecosystem by providing a robust, pipeline-friendly interface that handles the operational realities of large-scale programmatic querying. xgt is freely available under the MIT and Apache~2.0 dual licence at https://github.com/Ebedthan/xgt.
**Corresponding Author:**	Anicet E. T. Ebou, Ph.D.<br>Institut National Polytechnique Felix Houphouet-Boigny<br>Yamoussoukro, CÔTE D'IVOIRE
**Corresponding Author Secondary Information:**	
**Corresponding Author's Institution:**	Institut National Polytechnique Felix Houphouet-Boigny
**Corresponding Author's Secondary Institution:**	
**First Author:**	Anicet E. T. Ebou, Ph.D.
**First Author Secondary Information:**	
**Order of Authors:**	Anicet E. T. Ebou, Ph.D.
	Dominique K. Koua, Ph.D.
	Adolphe Zézé, Ph.D.
**Order of Authors Secondary Information:**	
**Response to Reviewers:**	Rebuttal 1<br><br>Dear Editor and Reviewers,

Powered by Editorial Manager® and ProduXion Manager® from Aries Systems Corporation

We thank the reviewers and the editorial team for their careful reading of our manuscript and for the constructive, precise, and on-point comments that have significantly strengthened the work. We have addressed all comments in full and provide a point-by-point response below. Page and line references correspond to the revised manuscript. All changes in the revised manuscript (Supplementary file in docx format) are highlighted in red.

We also confirm that xgt is registered in the SciCrunch database (RRID: SCR_028473) and in the bio.tools registry (bio.tools ID: xgt), as requested by the editorial team.

Reviewer 1

Comment R1.1: The biological validation is limited to one phylum and lacks ecological interpretation. The authors should explain why reclassifications occur and what the downstream implications are for GTDB users.

Response: We thank the reviewer for this important point. We have added a dedicated paragraph in the Discussion section that interprets the observed reclassification pattern in biological terms (Discussion page 4-5, paragraph 2, Lines 104-115). We explain that changes at the species (19.7%) and genus (15.6%) levels reflect the known dynamics of GTDB revision, in which most updates arise from the integration of newly sequenced genomes that refine species boundaries and reshape genus-level circumscriptions. We also note that reclassification at the family (14.1%) and order (10.3%) levels was substantial, indicating that phylogenetic restructuring extended beyond species circumscription. We further articulate three concrete downstream consequences: species-level assignments from published amplicon studies may refer to different phylogenetic entities in a newer release; MAG catalogues built against R214 may map reads to genera that were synonymised or split in R232; and cross-study comparisons that do not report the GTDB release version are inherently ambiguous. The revised paragraph concludes by framing xgt diff as a solution that makes release-aware taxonomy a tractable step in routine workflows.

Comment R1.2: The comparison is limited to curl+jq and python+requests. No comparison is made with workflow tools such as Snakemake or Nextflow, or with R packages such as taxize or rentrez.

Response: We thank the reviewer for this observation. We have added a paragraph in the Discussion (page 4-5, paragraph 3, Lines 116-127) that contextualises xgt relative to these tools. We clarify that Snakemake and Nextflow are workflow orchestration systems that can call xgt as a step within larger genomic pipelines but do not expose GTDB REST API functionality directly; similarly, R packages such as taxize provide NCBI-based taxonomy utilities rather than GTDB access. These tools are therefore better understood as composable partners than as alternatives to xgt. We explicitly note that none of them expose GTDB REST API functionality directly, and that xgt is designed to integrate with rather than replace existing pipeline infrastructure.

Reviewer 2

Comment R2.1 (major): TaxonKit with gtdb-taxdump is not discussed or compared against. The novelty claim for xgt diff is overstated. TaxonKit can also compute cross-release taxonomic changes.

Response: We thank the reviewer for this important correction. We have made three changes in response. First, we have added TaxonKit with gtdb-taxdump as a fourth column to the comparison table (Table S3, supplementary file). The table now includes rows for GTDB API queries, batch input, automatic pagination, retry with backoff, output formats, cross-release diff, local database requirement, accession-based input, runtime dependencies, and shell completions, with values for all four tools. TaxonKit correctly receives a checkmark for cross-release diff and correct entries reflecting that it requires a local database download and operates on taxon IDs rather than genome accessions.
Second, we have added a paragraph in the Discussion (paragraph 3, lines 116-123)

that directly compares xgt diff with TaxonKit + gtdb-taxdump, clearly articulating the differentiating characteristics: xgt diff operates against the live GTDB REST API without any local installation, accepts genome accessions as input, and returns complete taxonomy snapshots at both releases for contextual interpretation. We acknowledge that TaxonKit is preferable for large-scale offline batch analysis and explicitly frame the two tools as complementary rather than competing.
Third, we have softened the novelty claim in the opening sentence of the Discussion from "xgt diff is the first tool" to "to our knowledge, no existing tool provides a dedicated command-line interface for programmatic access to the GTDB REST API without requiring a local database installation, and xgt diff is, to our knowledge, the only tool that performs reproducible, scriptable cross-release taxonomic comparison operating directly on genome accessions via the live API." We have added the citation for TaxonKit (Shen and Ren, 2021, ref [8] of the revised manuscript).

Comment R2.2 (major): The 38.3% reclassification rate is computed over genomes common to both R214 and R232, not over all R232 representatives. The statistic is misrepresented and the denominator is unclear.

Response: The reviewer is precisely correct and we apologise for this imprecision. We have corrected both the Results and Discussion sections to clearly state the three relevant quantities: (1) the 46,827 R232 species representative genomes initially retrieved for p__Pseudomonadota; (2) the 26,112 (55.8%) absent from R214 (genomes added to GTDB between releases) that were excluded from comparison; and (3) the 20,715 genomes present in both releases that formed the analysis dataset, of which 7,941 (38.3%) were reclassified. We also note that the 55.8% exclusion rate itself is informative: it reflects a more than twofold increase in representative diversity within p__Pseudomonadota between R214 and R232, which is itself a noteworthy observation about the pace of GTDB expansion. The Discussion paragraph that repeats the 38.3% figure has been updated to include the correct denominator. The reclassification table caption (Table S2, supplementary) has also been updated to clarify that n = 20,715 refers to genomes present in both releases out of 46,827 R232 representatives. All changes can be viewed at lines 78-92, lines100-103 of the revised manuscript and in the caption of Table S2.

Comment R2.minor.1: Figure 1 shows 36,403 accessions for g__Escherichia but the text states 52,587. The figure appears to have been generated from an earlier GTDB release.

Response: The reviewer is correct. Figure 1 was generated when the GTDB release current at the time contained fewer Escherichia genomes. We have regenerated the search output panel of Figure 1 using the current GTDB API (release R232), which returns 52,403 accessions for g__Escherichia, consistent with the text.

Comment R2.minor.2: Figure 1 diff panel shows --from R212, which does not exist. This appears to be a typo for R214.

Response: The reviewer is correct. The diff panel was generated with an incorrect release flag. We have regenerated the diff panel using the correct command xgt diff GCA_000005845.2 --from R214 --to R232, and Figure 1 now shows the correct release identifier R214 in the command line.

Comment R2.minor.3: Figure 1 diff JSON output has a syntax error: the "to" key is missing its opening quotation mark.

Response: The reviewer is correct. The JSON displayed in the diff panel had a typographic error. This has been corrected in the regenerated Figure 1 (see response to R2.minor.2 above), which shows syntactically valid JSON output from the actual tool.

Comment R2.minor.4: Table S1 caption states that relative speedup is with respect to the slowest tool, but the values show xgt = 1.00 (i.e., relative to the fastest tool). The caption is incorrect.

Response: The reviewer is correct. The caption has been corrected to read "Relative

speedup is with respect to xgt (the fastest tool in each group)" in the revised supplementary file.

Comment R2.minor.5: The main text states 4.27× speedup for the medium query but Table S1 shows a different value. There is an inconsistency between mean-based and median-based ratios.

Response: We confirm that the 4.27× figure in the main text is median-based (xgt median 1.502 s, curl+jq median 6.412 s, ratio = 4.27), which is the correct statistic to report given the skewed distributions. The Relative column in Table S1 was based on mean values in the previous version, creating the apparent inconsistency. We have updated the Relative column in Table S1 to use median-based ratios throughout, ensuring full consistency between the table and the main text.

Comment R2.minor.6: The text discusses only species and genus reclassifications but Table S2 shows substantial changes also at the family and order levels.

Response: We agree that the order and family level changes deserved explicit discussion. This has been addressed in the new biological interpretation paragraph in the Discussion (see response to R1.1 above), which now states: "reclassification at the family (14.1%) and order (10.3%) levels was also substantial, indicating that phylogenetic restructuring extended well beyond species circumscription.". This change is visible at line 107 to 110.

Comment R2.minor.7: The gtdb_reclassification_analysis.sh script fails with an undefined variable error when run with --limit 20.

Response: We thank the reviewer for testing the script. The bug was traced to two issues: (1) the banner line contained a Unicode arrow character (→, U+2192) that caused an encoding error under set -euo pipefail in shell environments using non-UTF-8 locales, causing the script to exit silently before the --limit logic was reached; and (2) the TOTAL_ACC variable computed by wc -l was not robustly stripped of all whitespace variants (tabs and newlines in addition to spaces) on all platforms. Both issues have been fixed. The fixed script is available in the repository at https://github.com/Ebedthan/xgt_article.

We hope that the revised manuscript and this point-by-point response adequately address all concerns raised. We remain available to provide any further information or clarification the editorial team may require.

Editorial Team

Comment Editor.1: The authors are encouraged to cite relevant GigaScience papers, particularly GFFx, a Rust-based genomic utility suite also published in GigaScience.

Response: We thank the editorial team for this suggestion. We have added a citation to GFFx (Chen et al., 2025, GigaScience, ref [6] of the revised manuscript) at the point in the introduction where xgt's implementation in Rust is first justified, specifically in the sentence describing Rust as "a systems programming language increasingly adopted for high-performance bioinformatics tools." This citation directly supports that claim and acknowledges prior work in the same venue.

Comment Editor.2: Please register the tool in bio.tools and in SciCrunch to obtain an RRID.

Response: Both registrations are complete. xgt is registered in the SciCrunch database (RRID: SCR_028473) and in the bio.tools registry (bio.tools ID: xgt). Both identifiers appear in the Availability section of the revised manuscript.

Yours sincerely,

Anicet E. T. Ebou (corresponding author)
ediman.ebou@inphb.ci

Powered by Editorial Manager® and ProduXion Manager® from Aries Systems Corporation

	On behalf of all authors:<br>Dominique K. Koua<br>Adolphe Zézé<br><br>Laboratoire de Microbiologie, Biotechnologie et Bioinformatique<br>Institut National Polytechnique Félix Houphouët-Boigny<br>BP 1093 Yamoussoukro, Côte d'Ivoire
**Additional Information:**	
**Question**	**Response**
Are you submitting this manuscript to a special series or article collection?	No
**Experimental design and statistics**<br><br>Full details of the experimental design and statistical methods used should be given in the Methods section, as detailed in our Minimum Standards Reporting Checklist. Information essential to interpreting the data presented should be made available in the figure legends.<br><br>Have you included all the information requested in your manuscript?	Yes
**Resources**<br><br>A description of all resources used, including antibodies, cell lines, animals and software tools, with enough information to allow them to be uniquely identified, should be included in the Methods section. Authors are strongly encouraged to cite Research Resource Identifiers (RRIDs) for antibodies, model organisms and tools, where possible.<br><br>Have you included the information requested as detailed in our Minimum Standards Reporting Checklist?	Yes
**Availability of data and materials**<br><br>All datasets and code on which the conclusions of the paper rely must be either included in your submission or	Yes

Powered by Editorial Manager® and ProduXion Manager® from Aries Systems Corporation

deposited in publicly available repositories (where available and ethically appropriate), referencing such data using a unique identifier in the references and in the “Availability of Data and Materials” section of your manuscript.<br><br>Have you have met the above requirement as detailed in our Minimum Standards Reporting Checklist?	
GigaScience has policies and guidelines in place for the use of generative AI-writing tools such as ChatGPT. If you have used such writing tools to assist with writing the manuscript this must be declared and cited in the text. Authors should not list AI-writing tools and other AI-assisted technologies as an author or co-author and should acknowledge that they are fully responsible for text generated or refined by AI-writing tools.<p><br><br>A summary of use (particularly in the introduction or among methods) needs to be included at the end of the paper, and the outputs should also be included as a supplementary file hosted in GigaDB or other open repositories. Please read our guidelines for more information. <p><br><br>By submitting to GigaScience, you are aware of the journal's AI-writing tools policy, and if you have declared use of such tools below, you have acknowledged this where appropriate in your manuscript and have made a summary of use and outputs available. <p> <b>AI-assisted writing tools have been used in the preparation of this manuscript?	No

Manuscript

Click here to access/download;Manuscript;brief_communication_R1.pdf

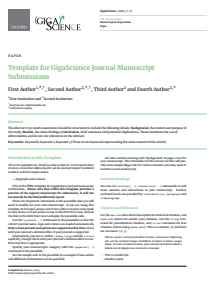

GigaScience, 2026, 1–4

doi: xx.xxxx/xxxx
Manuscript in Preparation
Brief Communication

BRIEF COMMUNICATION

xgt: a command-line interface for the Genome Taxonomy Database with cross-release taxonomic comparison

Anicet E. T. Ebou[1,*], Dominique K. Koua[1] and Adolphe Zézé[1]

[1]Laboratoire de Microbiologie, Biotechnologie et Bioinformatique, Institut National Polytechnique Félix Houphouët-Boigny, BP 1093 Yamoussoukro, Côte d'Ivoire

*ediman.ebou@inphb.ci

Key words: GTDB; Microbial taxonomy; Genomic database; Taxonomic comparison; Command-line tool; Rust.

Background

The Genome Taxonomy Database (GTDB) provides a standardised, phylogenetically informed classification of bacterial and archaeal genomes [1], and has become the reference taxonomy in microbial ecology, comparative genomics, and environmental sequencing [2, 3]. GTDB releases are versioned (e.g. R214, R220, R232), and taxonomic assignments can change substantially between releases as new genomes are incorporated and phylogenetic placements are revised, directly affecting the reproducibility of studies conducted across release periods.

Despite exposing a REST API, GTDB provides no dedicated command-line interface. Researchers querying multiple accessions, performing batch searches, or tracking taxonomic changes across releases must write custom scripts or rely on general-purpose tools such as `curl`. Such tools requires manual JSON parsing, provide no pagination, and offer no retry logic. `GTDB-Tk` [4] assigns taxonomy to new assemblies using a local reference tree but does not query the REST API. To our knowledge, no published tool provides a dedicated command-line interface for programmatic GTDB access covering batch retrieval, release-specific queries, and cross-release taxonomic comparison.

Here we present `xgt`, an open-source command-line tool written in Rust [5] , a systems programming language increasingly adopted for high-performance bioinformatics tools [6], that provides efficient, reproducible access to the GTDB REST API through four subcommands: `search`, `genome`, `taxon`, and `diff`. `xgt` is compiled as a self-contained binary with no runtime dependencies, available for Linux, macOS, and Windows at https://github.com/Ebedthan/xgt.

Results

`xgt` exposes four subcommands through a unified interface (Figure 1):

```
xgt <subcommand> [OPTIONS]
  ↪ [QUERY|ACCESSION|NAME]
```

All subcommands accept a single query as a positional argument, a file via `--file`, or standard input via `--file -`, enabling direct use in Unix pipelines. Output is controlled by `--outfmt csv|tsv|json` and can be directed to a single file (`--out`) or split into one file per query (`--split`). A progress bar is displayed on standard error during batch runs, preserving stdout for downstream tools.

`xgt search` queries GTDB for genomes by name, accession, or taxonomy string with exact or partial matching, and returns results as CSV, TSV, or JSON. Pagination is transparent: `xgt` fetches all result pages sequentially, ensuring that queries on large genera (*g__Escherichia*, over 52 000 genomes) complete without truncation.

`xgt genome` retrieves full genome cards (nucleotide statistics, CheckM/CheckM2 quality metrics, NCBI metadata, GTDB classification), lightweight metadata, or complete taxonomic histories via mutually exclusive flags (`--metadata`, `--history`). The `--release` flag targets a specific GTDB release for card and metadata queries.

`xgt taxon` retrieves taxonomic records for named GTDB taxa, lists associated genome accessions (`--genomes`), or searches for taxon names in the current or all historical GTDB releases (`--search`, `--all`).

`xgt diff` addresses the principal gap in existing GTDB tooling. To our knowledge, no previously published tool computes per-rank taxonomic changes between arbitrary GTDB release pairs. `xgt diff` queries the existing `/genome/{accession}/taxon-history` endpoint

Compiled on: July 22, 2026.
Draft manuscript prepared by the author.

and compares the seven-rank GTDB classification at a source release (`--from`) and a target release (`--to`; defaults to the most recent available). For each accession, the output reports a `changed` flag, per-rank change records carrying the old and new assignment, and full taxonomy snapshots at both releases. For example, comparing `GCA_000005845.2` between R214 and R232 reveals a species-level reclassification from `s__Escherichia coli` to `s__G047199095 sp047199095`.

Performance

We benchmarked `xgt` against `curl` + `jq` and `python` + `requests` on the `search --id` task across three genus sizes using `hyperfine` 1.20.0 [7] (10 timed runs, 3 warmup runs; full protocol in Supplementary Methods). `xgt` was consistently fastest (Table S1). For the medium query (*g__Bacillus*, 14 API pages), the median completion time was 1.50 s versus 6.41 s for `curl` + `jq` (4.27× faster), reflecting per-page process invocation overhead. For the large query (*g__Escherichia*, 53 pages), network jitter dominated; the median times of 5.3, 13.1, and 25.2 s for `xgt`, `python`, and `curl` + `jq` respectively should be interpreted as real-world workflow efficiency rather than isolated computational benchmarks (Table S1).

Biological validation

To demonstrate the biological utility of `xgt diff`, we retrieved the 46,827 R232 species representative genomes assigned to `p__Pseudomonadota` using `xgt search`. Of these, 26 112 (55.8%) were absent from R214 and excluded from comparison, reflecting the substantial expansion of the `p__Pseudomonadota` genome set between the two releases, a more than twofold increase in representative diversity within this phylum alone. Of the remaining 20 715 genomes present in both releases, 7 941 (38.3%) were reclassified at one or more ranks (Table S2). Changes were concentrated at the species (19.7%) and genus (15.6%) levels, with higher ranks largely stable, consistent with the known dynamics of GTDB revision in which most updates reflect refined species circumscriptions and genus-level phylogenetic restructuring rather than changes to higher-rank framework taxa. A subset of 3 636 genomes (45.8% of reclassified) changed at two or more ranks simultaneously, indicating genuine phylogenetic repositioning.

These results illustrate that even between consecutive GTDB releases, a non-trivial proportion of reference genomes receive updated assignments that would silently affect taxonomic profiles, ecological comparisons, and database cross-references if release versions are not tracked explicitly. `xgt diff` provides a reproducible, single-command approach to this audit for any user-defined genome set.

Discussion

`xgt` fills a practical gap in the GTDB ecosystem: to our knowledge, no existing tool provides a dedicated command-line interface for programmatic access to the GTDB REST API without requiring a local database installation, and `xgt diff` is, to our knowledge, the only tool that performs reproducible, scriptable cross-release taxonomic comparison operating directly on genome accessions via the live API. The biological validation demonstrates that reclassification between GTDB releases is not rare (38.3% of the 20 715 `p__Pseudomonadota` representatives present in both R214 and R232 changed between releases) with direct consequences for the reproducibility of amplicon, MAG, and reference database workflows.

The reclassification pattern observed in `p__Pseudomonadota` reflects the known dynamics of GTDB revision: most changes between releases arise from the integration of newly sequenced genomes that refine species boundaries and reshape genus-level circumscriptions, rather than from fundamental changes to higher-rank framework taxa. The concentration of changes at the species (19.7%) and genus (15.6%) levels is consistent with this, though reclassification at the family (14.1%) and order (10.3%) levels was also substantial, indicating that phylogenetic restructuring extended well beyond species circumscription. For downstream analyses, these changes have concrete consequences: species-level assignments from a published amplicon study may refer to different phylogenetic entities in a newer release; MAG catalogues built against R214 may map reads to genera that were synonymised or split in R232; and cross-study comparisons that do not report the GTDB release version are inherently ambiguous. `xgt diff` makes release-aware taxonomy a tractable step in routine workflows rather than a manual post-processing burden.

Several tools offer complementary functionality worth distinguishing from `xgt`. `TaxonKit` with `gtdb-taxdump` [8] can compute taxonomic changes between GTDB releases by diffing locally downloaded taxonomy dumps. This approach is well suited to bulk offline analysis but requires downloading and maintaining local database files of several gigabytes, and operates on taxon IDs rather than genome accessions. `xgt diff` operates directly against the live GTDB REST API without any local installation, accepts genome accessions as input, and returns complete taxonomy snapshots at both releases for contextual interpretation. The two approaches are complementary: `TaxonKit` is preferable for large-scale offline batch analysis, while `xgt` is suited to interactive use and integration into API-dependent pipelines. Workflow management systems such as Snakemake [9] and Nextflow [10] can orchestrate `xgt` as a step within larger genomic pipelines, and R packages such as `taxize` provide NCBI-based taxonomy utilities; none of these tools expose GTDB REST API functionality directly, and they are better understood as composable partners than as alternatives to `xgt`.

Current limitations include sequential pagination (throughput bounded by per-page API latency), API-dependent operation (no offline mode), and partial release filtering (the `--release` flag applies only to genome card and metadata endpoints; `search` and `taxon` endpoints always return the current release). Future development will introduce tolerant deserialisation to handle API type changes without tool updates, and parallel pagination with rate-limit management. As GTDB continues to expand, from approximately 258 000 genomes in R207 to over 400 000 in R232 [1], scalable, release-aware programmatic access will become increasingly important. `xgt` is designed to grow with the database and serve as a stable foundation for GTDB-dependent workflows.

Availability of supporting source code and requirements

- **Project name:** xgt
- **Project home page:** https://github.com/Ebedthan/xgt
- **Operating system(s):** Linux (x86_64), macOS (x86_64, Apple Silicon), Windows (x86_64)
- **Programming language:** Rust (edition 2021, MSRV 1.85)
- **Other requirements:** None (self-contained binary); Rust 1.85 or later required to build from source
- **License:** MIT and Apache 2.0 (dual licence)
- **RRID:** SCR_028473
- **bio.tools ID:** xgt

Benchmark and biological validation scripts: https://github.com/Ebedthan/xgt_article.

Abbreviations

API: Application Programming Interface; **CLI**: Command-Line Interface; **CSV**: Comma-Separated Values; **GTDB**: Genome Taxonomy

Database; **JSON**: JavaScript Object Notation; **MAG**: Metagenome-Assembled Genome; **REST**: Representational State Transfer; **TLS**: Transport Layer Security; **TSV**: Tab-Separated Values.

Consent for publication

Not applicable

Competing Interests

The authors declare no competing interest.

Funding

Not applicable.

Author's Contributions

Anicet E. T. Ebou: Conceptualization, Methodology, Software, Writing - Original draft. **Dominique K. Koua**: Writing - Review & editing, Methodology, Conceptualization. **Adolphe Zézé**: Writing - Review & editing, Supervision.

Acknowledgements

Not applicable.

References

1. Parks DH, Chuvochina M, Rinke C, Mussig AJ, Chaumeil PA, Hugenholtz P. GTDB: An Ongoing Census of Bacterial and Archaeal Diversity through a Phylogenetically Consistent, Rank Normalized and Complete Genome-Based Taxonomy. Nucleic Acids Research 2022 Jan;50(D1):D785–D794.
2. Riesco R, Trujillo ME. Update on the Proposed Minimal Standards for the Use of Genome Data for the Taxonomy of Prokaryotes. International Journal of Systematic and Evolutionary Microbiology 2024;74(3):006300.
3. Clagnan E, Costanzo M, Visca A, Di Gregorio L, Tabacchioni S, Colantoni E, et al. Culturomics- and Metagenomics-Based Insights into the Soil Microbiome Preservation and Application for Sustainable Agriculture. Frontiers in Microbiology 2024 Oct;15.
4. Chaumeil PA, Mussig AJ, Hugenholtz P, Parks DH. GTDB-Tk: A Toolkit to Classify Genomes with the Genome Taxonomy Database. Bioinformatics 2020 Mar;36(6):1925–1927.
5. Klabnik S, Nichols C. The Rust Programming Language. No Starch Press; 2018.
6. Chen B, Wu D, Zhang G. GFFx: A Rust-based Suite of Utilities for Ultra-Fast Genomic Feature Extraction. GigaScience 2025 Jan;14:giaf124.
7. Peter D, hyperfine: A command-line benchmarking tool; 2023. https://github.com/sharkdp/hyperfine.
8. Shen W, Ren H. TaxonKit: A Practical and Efficient NCBI Taxonomy Toolkit. Journal of Genetics and Genomics 2021 Sep;48(9):844–850.
9. Mölder F, Jablonski KP, Letcher B, Hall MB, van Dyken PC, Tomkins-Tinch CH, et al., Sustainable Data Analysis with Snakemake. F1000Research; 2025.
10. Di Tommaso P, Chatzou M, Floden EW, Barja PP, Palumbo E, Notredame C. Nextflow Enables Reproducible Computational Workflows. Nature Biotechnology 2017 Apr;35(4):316–319.

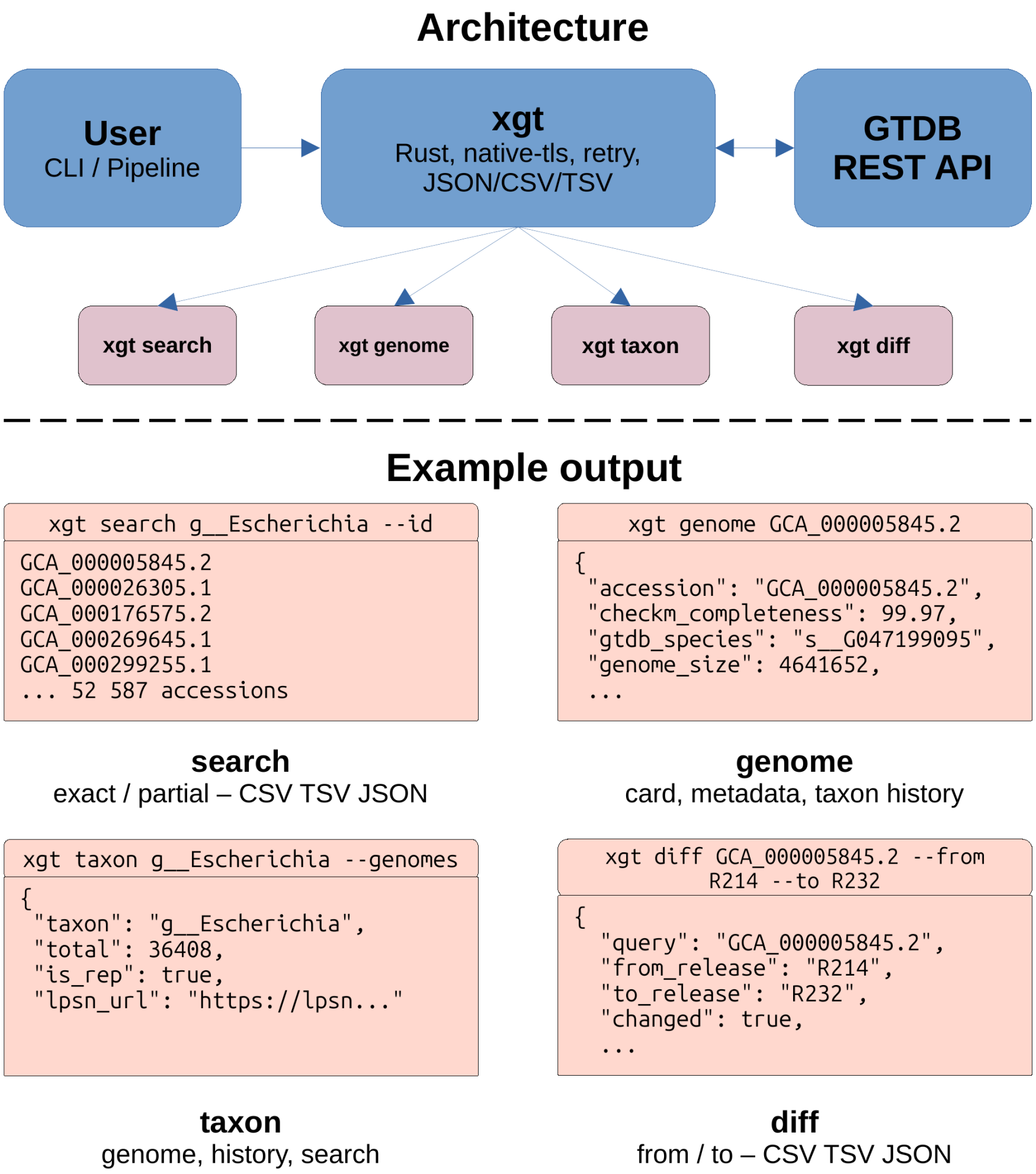

Figure 1. Overview of `xgt` architecture, subcommands, and representative outputs. The upper panel shows the three-component architecture: the user interacts with `xgt` via the command line or a pipeline; `xgt` communicates bidirectionally with the GTDB REST API using a blocking HTTP client with native TLS, automatic retry, and support for JSON, CSV, and TSV output. Four subcommands are exposed: `search`, `genome`, `taxon`, and `diff`. The lower panels show representative outputs for each subcommand. Output fields are truncated for display.

Figure

Click here to access/download;Figure;fig1_R1.png

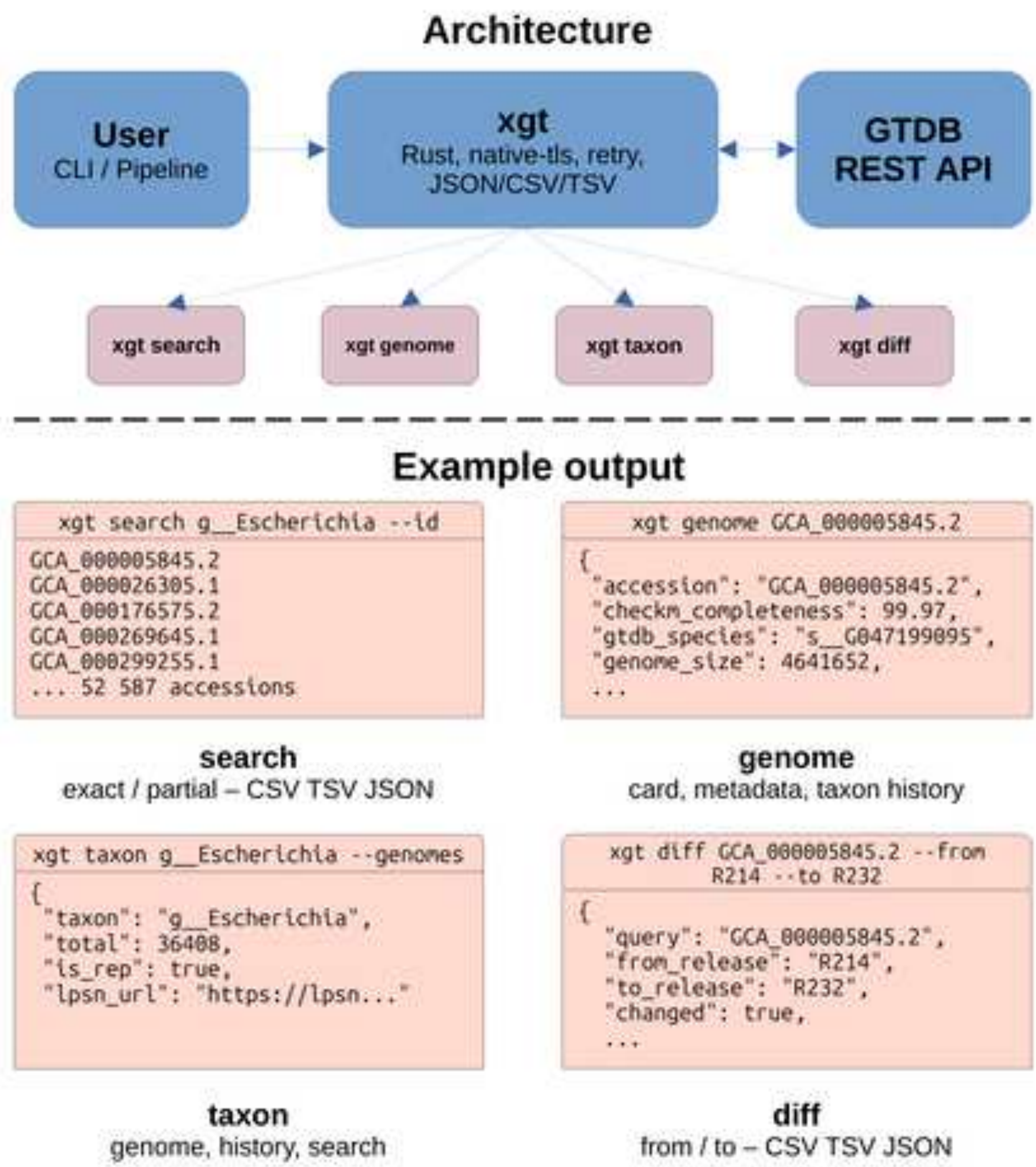

Click here to access/download
Supplementary Material
supplementary_R1.pdf

Manuscript in docx format with line number and track changes

Click here to access/download
Supplementary Material
xgt_article_R1.docx
