# Supplementary material for: xgt: a command-line interface for the Genome Taxonomy Database with cross-release taxonomic comparison
Source: Gigascience. 2026 Aug 14;15:giag086. doi: 10.1093/gigascience/giag086 (PMC13505384; doi:10.1093/gigascience/giag086)
Supplement: Supplementary file 3 [file giag086_giga-d-26-00216_revision_2.pdf]

GigaScience

xgt: a command-line interface for the Genome Taxonomy Database with cross-release taxonomic comparison

--Manuscript Draft--

Manuscript Number:	GIGA-D-26-00216R2
Full Title:	xgt: a command-line interface for the Genome Taxonomy Database with cross-release taxonomic comparison
Article Type:	Brief Communication
Funding Information:	
Abstract:	Background:<br>The Genome Taxonomy Database (GTDB) provides a standardised, phylogenetically informed classification of bacterial and archaeal genomes, widely used as the reference taxonomy in microbial ecology and comparative genomics. Despite exposing a REST API, GTDB lacks a dedicated command-line interface, leaving researchers to write custom scripts that lack pagination for large result sets, retry logic for transient failures, and support for tracking taxonomic reclassifications across release versions.<br>Findings:<br>We present xgt, an open-source command-line tool written in Rust that provides efficient, flexible access to the GTDB REST API through four subcommands. xgt search queries GTDB for genomes by name, accession, or taxonomy with exact or partial matching. xgt genome retrieves full genome cards, lightweight metadata, or complete taxonomic histories. xgt taxon explores taxon lineages and associated genome sets. xgt diff computes per-rank taxonomic changes for a genome between any two GTDB releases, a capability not previously available in a dedicated GTDB command-line tool, to our knowledge. All subcommands support batch processing from files or standard input, transparent pagination for arbitrarily large result sets, structured output in JSON, CSV, and TSV formats, per-item file splitting for pipeline integration, and automatic retry with exponential backoff. xgt is compiled as a self-contained binary, with no external runtime dependencies, available for Linux, macOS, and Windows.<br>Conclusions:<br>xgt fills a practical gap in the GTDB ecosystem by providing a robust, pipeline-friendly interface that handles the operational realities of large-scale programmatic querying. xgt is freely available under the MIT and Apache~2.0 dual licence at https://github.com/Ebedthan/xgt.
Corresponding Author:	Anicet E. T. Ebou, Ph.D.<br>Institut National Polytechnique Felix Houphouet-Boigny<br>Yamoussoukro, CÔTE D'IVOIRE
Corresponding Author Secondary Information:	
Corresponding Author's Institution:	Institut National Polytechnique Felix Houphouet-Boigny
Corresponding Author's Secondary Institution:	
First Author:	Anicet E. T. Ebou, Ph.D.
First Author Secondary Information:	
Order of Authors:	Anicet E. T. Ebou, Ph.D.
	Dominique K. Koua, Ph.D.
	Adolphe Zézé, Ph.D.
Order of Authors Secondary Information:	
Response to Reviewers:	Rebuttal Round 2<br><br>Dear Editor and Reviewers,

Powered by Editorial Manager® and ProduXion Manager® from Aries Systems Corporation

We thank the reviewers for their continued careful reading of the manuscript and for the positive assessment of our revision. We are pleased that Reviewer 1 has no remaining concerns. We have addressed all four points raised by Reviewer 2, each of which identified a genuine inconsistency between the revised sections and sections that were not updated in the previous round. All changes are highlighted in red across (xgt_article_R2.docx) the updated manuscript and we provide a point-by-point response below.

Reviewer 2

Comment R2.1 (minor): The novelty claim has been softened in the Discussion, but the Results section still reads "To our knowledge, no previously published tool computes per-rank taxonomic changes between arbitrary GTDB release pairs", which is unchanged from the first version. The new Discussion paragraph acknowledges TaxonKit with gtdb-taxdump, so the two sections now contradict each other.

Response: The reviewer is correct that we softened the claim in the Discussion but overlooked the same sentence in the Results section. We have now updated the Results sentence to match the Discussion wording. The revised sentence reads: "To our knowledge, no previously published tool provides a dedicated command-line interface to compute per-rank taxonomic changes between arbitrary GTDB release pairs directly via the live API without requiring a local database installation." This formulation is consistent with the Discussion paragraph that acknowledges TaxonKit with gtdb-taxdump as a tool that can compute cross-release changes from locally downloaded taxonomy dumps, and correctly identifies the specific niche that xgt diff fills. The changes are visible from lines 57 to 69.

Comment R2.2 (minor): The Results and Discussion now report the three quantities correctly, but Supplementary Methods S1.6 was not updated. It still describes the analysis as covering "species representative genomes assigned to p__Pseudomonadota" with no counts and no note that xgt search returns the current release. S1.6 is where a reader will go for the cohort definition.

Response: We thank the reviewer for catching this omission. We have revised S1.6 to match the main text in full. The updated paragraph now states explicitly that: (1) 46,827 R232 species representative genomes were initially retrieved; (2) the search and taxon endpoints always return data from the current GTDB release, so the retrieval corresponds to the representative set as it stood on 25 May 2026 (R232); (3) 26,112 accessions (55.8%) were absent from R214 and excluded; and (4) the remaining 20,715 genomes present in both releases formed the analysis dataset. The retrieval command and all subsequent steps are unchanged.

Comment R2.3 (minor): The search panel of Figure 1 now shows 52,587 accessions but the taxon panel still shows "total": 36408, so the figure now shows two different numbers for g__Escherichia. Additionally, neither number is the true size of the genus: xgt search g__Escherichia --field gtdb returns 37,464, and the default search also matches the NCBI taxonomy field. The 36,408 in the taxon panel is the API total for s__Escherichia coli, not the genus. The Results text should also be reworded accordingly.

Response: The reviewer is correct on all counts. We had regenerated only the search panel and overlooked the taxon panel, which displayed the result for "s__Escherichia coli" rather than g__Escherichia. We have regenerated the taxon panel using xgt taxon g__Escherichia against the current API, and both panels now show consistent values for the same entity.
We also thank the reviewer for the careful observation that 52,587 is the number of records the default search returns by matching both GTDB and NCBI taxonomy fields, not the number of genomes in the genus itself (~37,000 by GTDB taxonomy). We have reworded the sentence in the Results section (Lines 48-49) from "queries on large genera like Escherichia (52,000+ genomes)" to "queries that return large result sets (for example, a search matching 52,000+ records)" to avoid attributing a specific genome count to the genus. The benchmark description is unaffected since it correctly refers to the number of accessions returned by the query (52,587) rather than the

Powered by Editorial Manager® and ProduXion Manager® from Aries Systems Corporation

	genus size.<br><br>Comment R2.4 (minor): The Discussion states that GTDB grew "from approximately 258,000 genomes in R207 to over 400,000 in R232". The published GTDB statistics give 317,542 for R207 and 901,341 for R232. The figures in the manuscript correspond to R202 and R214 respectively, so both are two releases out of date.<br><br>Response: The reviewer is correct. We apologise for this error. The figures 258,000 and 400,000 correspond to GTDB releases R202 and R214 respectively, not R207 and R232. We have corrected the sentence to read "from approximately 317,000 genomes in R207 to over 901,000 in R232", using the figures from the published GTDB release statistics. We note that the reviewer also correctly points out that this correction strengthens the sentence: the growth between R207 and R232 is approximately threefold, which is a more compelling illustration of the expanding scale of the database than the 1.5-fold implied by the previous (incorrect) figures.<br><br>We believe the revised manuscript now fully addresses all reviewer concerns and contains no remaining inconsistencies between sections.<br><br>Yours sincerely,<br><br>Anicet E. T. Ebou (corresponding author)<br>ediman.ebou@inphb.ci<br><br>On behalf of all authors:<br>Dominique K. Koua<br>Adolphe Zézé<br><br>Laboratoire de Microbiologie, Biotechnologie et Bioinformatique<br>Institut National Polytechnique Félix Houphouët-Boigny<br>BP 1093 Yamoussoukro, Côte d'Ivoire
**Additional Information:**	
**Question**	**Response**
Are you submitting this manuscript to a special series or article collection?	No
**Experimental design and statistics**<br><br>Full details of the experimental design and statistical methods used should be given in the Methods section, as detailed in our Minimum Standards Reporting Checklist. Information essential to interpreting the data presented should be made available in the figure legends.<br><br>Have you included all the information requested in your manuscript?	Yes
**Resources**<br><br>A description of all resources used, including antibodies, cell lines, animals	Yes

and software tools, with enough information to allow them to be uniquely identified, should be included in the Methods section. Authors are strongly encouraged to cite Research Resource Identifiers (RRIDs) for antibodies, model organisms and tools, where possible.<br><br>Have you included the information requested as detailed in our Minimum Standards Reporting Checklist?	
**Availability of data and materials**<br><br>All datasets and code on which the conclusions of the paper rely must be either included in your submission or deposited in publicly available repositories (where available and ethically appropriate), referencing such data using a unique identifier in the references and in the "Availability of Data and Materials" section of your manuscript.<br><br>Have you have met the above requirement as detailed in our Minimum Standards Reporting Checklist?	Yes
GigaScience has policies and guidelines in place for the use of generative AI-writing tools such as ChatGPT. If you have used such writing tools to assist with writing the manuscript this must be declared and cited in the text. Authors should not list AI-writing tools and other AI-assisted technologies as an author or co-author and should acknowledge that they are fully responsible for text generated or refined by AI-writing tools.<p><br><br>A summary of use (particularly in the introduction or among methods) needs to be included at the end of the paper, and the outputs should also be included as a supplementary file hosted in GigaDB or other open repositories. Please read our guidelines for more information. <p><br><br>By submitting to GigaScience, you are	No

aware of the journal's AI-writing tools policy, and if you have declared use of such tools below, you have acknowledged this where appropriate in your manuscript and have made a summary of use and outputs available. <p> <b>AI-assisted writing tools have been used in the preparation of this manuscript?	

Manuscript Click here to access/download;Manuscript;xgt_article_final.docx

xgt: a command-line interface for the Genome Taxonomy Database with cross-release taxonomic comparison

Anicet E. T. Ebou[1,*], Dominique K. Koua[1], Adolphe Zézé[1]

1. Laboratoire de Microbiologie, Biotechnologie et Bioinformatique, Institut National Polytechnique National Félix Houphouët-Boigny, BP 1093 Yamoussoukro, Côte d'Ivoire.

*Correspondence: Anicet E. T. Ebou, ediman.ebou@inphb.ci

Anicet E.T. Ebou [0000-0003-4005-177X]; Dominique K. Koua [0000-0002-9078-8844]; Adolphe Zézé [0009-0004-5941-6657].

Background

The Genome Taxonomy Database (GTDB) provides a standardised, phylogenetically informed classification of bacterial and archaeal genomes [1], and has become the reference taxonomy in microbial ecology, comparative genomics, and environmental sequencing [2,3]. GTDB releases are versioned (e.g. R214, R220, R232), and taxonomic assignments can change substantially between releases as new genomes are incorporated and phylogenetic placements are revised, directly affecting the reproducibility of studies conducted across release periods.

Despite exposing a REST API, GTDB provides no dedicated command-line interface. Researchers querying multiple accessions, performing batch searches, or tracking taxonomic changes across releases must write custom scripts or rely on general-purpose tools such as `curl`. Such tools require manual JSON parsing, provide no pagination, and offer no retry logic. GTDB-Tk [4] assigns taxonomy to new assemblies using a local reference tree but does not query the REST API. To our knowledge, no published tool provides a dedicated command-line interface for programmatic GTDB access covering batch retrieval, release-specific queries, and cross-release taxonomic comparison.

Here we present `xgt`, an open-source command-line tool written in Rust [5], a systems programming language increasingly adopted for high-performance bioinformatics tools [6], that provides efficient, reproducible access to the GTDB REST API through four subcommands: `search`, `genome`, `taxon`, and `diff`. `xgt` is compiled as a self-contained binary with no runtime dependencies, available for Linux, macOS, and Windows at https://github.com/Ebedthan/xgt.

Results

`xgt` exposes four subcommands through a unified interface (Figure 1):

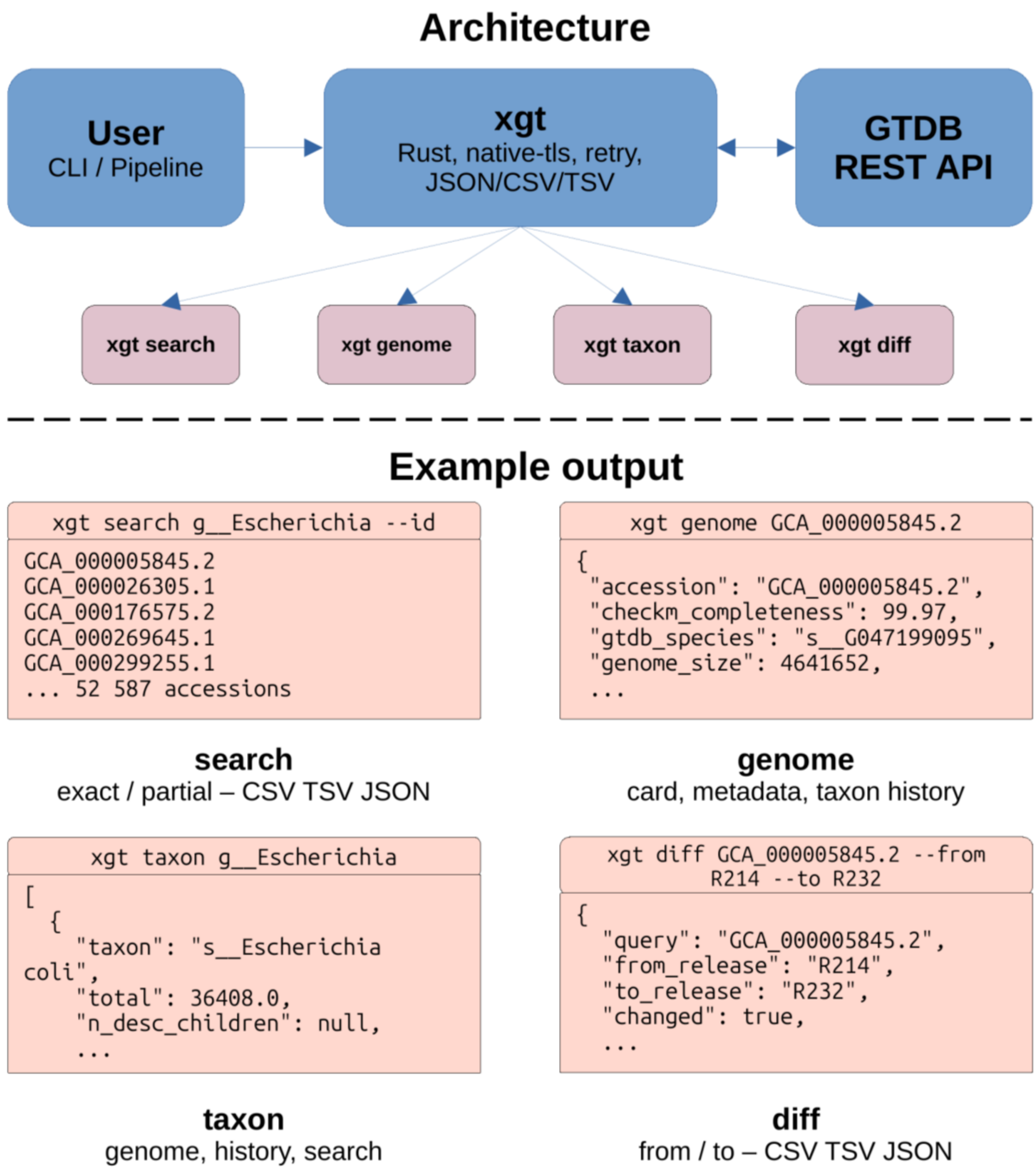

Figure 1. Overview of `xgt` architecture, subcommands, and representative outputs. The upper panel shows the three-component architecture: the user interacts with `xgt` via the command-line or a pipeline; `xgt` communicates bidirectionally with the GTDB REST API using a blocking HTTP client with native TLS, automatic retry, and support for JSON, CSV, and TSV output. Four subcommands are exposed: search, genome, taxon, and diff. The lower panels show representative outputs for each subcommand. Output fields are truncated for display.

```
xgt <subcommand> [OPTIONS] [QUERY|ACCESSION|NAME]
```

All subcommands accept a single query as a positional argument, a file via `--file`, or standard input via `--file -`, enabling direct use in Unix pipelines. Output is controlled by `--outfmt`

csv|tsv|json and can be directed to a single file (--out) or split into one file per query (--split).

A progress bar is displayed on standard error during batch runs, preserving stdout for downstream tools.

xgt search queries GTDB for genomes by name, accession, or taxonomy string with exact or partial matching, and returns results as CSV, TSV, or JSON. Pagination is transparent: xgt fetches all result pages sequentially, ensuring that queries that return large result sets (for example, a search matching 52 000+ records) return complete results without truncation.

xgt genome retrieves full genome cards (nucleotide statistics, CheckM/CheckM2 quality metrics, NCBI metadata, GTDB classification), lightweight metadata, or complete taxonomic histories via mutually exclusive flags (--metadata, --history). The --release flag targets a specific GTDB release for card and metadata queries.

xgt taxon retrieves taxonomic records for named GTDB taxa, lists associated genome accessions (--genomes), or searches for taxon names in the current or all historical GTDB releases (--search, --all).

xgt diff addresses the principal gap in existing GTDB tooling. To our knowledge, no previously published tool provides a dedicated command-line interface to compute per-rank taxonomic changes between arbitrary GTDB release pairs directly via the live API without requiring a local database installation. xgt diff queries the existing /genome/{accession}/taxon-history endpoint and compares the seven-rank GTDB classification at a source release (--from) and a target release (--to; defaults to the most recent available). For each accession, the output reports a changed flag, per-rank change records carrying the old and new assignment, and full taxonomy snapshots at both releases. For example, comparing GCA_000005845.2 between R214 and R232 reveals a species-level reclassification from s__Escherichia coli to s__G047199095 sp047199095.

Performance

We benchmarked xgt against curl + jq and python + requests on the search --id task across three genus sizes using hyperfine v1.20.0 [7] (10 timed runs, 3 warmup runs; full protocol in Supplementary Methods). xgt was consistently fastest (Table S1). For the medium query (g__Bacillus, 14 API pages), the median completion time was 1.50s versus 6.41s for curl + jq (4.27x faster), reflecting per-page process invocation overhead. For the large query (g__Escherichia, 53 pages), network jitter dominated; the median times of 5.3, 13.1, and 25.2s for xgt, python, and curl + jq respectively should be interpreted as real-world workflow efficiency rather than isolated computational benchmarks (Table S1).

Biological validation

To demonstrate the biological utility of xgt diff, we retrieved the 46,827 R232 species representative genomes assigned to p__Pseudomonadota using xgt search. Of these, 26,112 (55.8%) were absent from R214 and excluded from comparison, reflecting the substantial expansion

of the p__Pseudomonadota genome set between the two releases, a more than twofold increase in representative diversity within this phylum alone. Of the remaining 20,715 genomes present in both releases, 7,941 (38.3%) were reclassified at one or more ranks (Table S2). Changes were concentrated at the species (19.7%) and genus (15.6%) levels, with higher ranks largely stable, consistent with the known dynamics of GTDB revision in which most updates reflect refined species circumscriptions and genus-level phylogenetic restructuring rather than changes to higher-rank framework taxa. A subset of 3,636 genomes (45.8% of reclassified) changed at two or more ranks simultaneously, indicating genuine phylogenetic repositioning.

These results illustrate that even between consecutive GTDB releases, a non-trivial proportion of reference genomes receive updated assignments that would silently affect taxonomic profiles, ecological comparisons, and database cross-references if release versions are not tracked explicitly. `xgt diff` provides a reproducible, single-command approach to this audit for any user-defined genome set.

Discussion

`xgt` fills a practical gap in the GTDB ecosystem: to our knowledge, no existing tool provides a dedicated command-line interface for programmatic access to the GTDB REST API without requiring a local database installation, and `xgt diff` is, to our knowledge, the only tool that performs reproducible, scriptable cross-release taxonomic comparison operating directly on genome accessions via the live API. The biological validation demonstrates that reclassification between GTDB releases is not rare (38.3% of the 20,715 p__Pseudomonadota representatives present in both R214 and R232 changed between releases) with direct consequences for the reproducibility of amplicon, MAG, and reference database workflows.

The reclassification pattern observed in p__Pseudomonadota reflects the known dynamics of GTDB revision: most changes between releases arise from the integration of newly sequenced genomes that refine species boundaries and reshape genus-level circumscriptions, rather than from fundamental changes to higher-rank framework taxa. The concentration of changes at the species (19.7%) and genus (15.6%) levels is consistent with this, though reclassification at the family (14.1%) and order (10.3%) levels was also substantial, indicating that phylogenetic restructuring extended well beyond species circumscription. For downstream analyses, these changes have concrete consequences: species-level assignments from a published amplicon study may refer to different phylogenetic entities in a newer release; MAG catalogues built against R214 may map reads to genera that were synonymised or split in R232; and cross-study comparisons that do not report the GTDB release version are inherently ambiguous. `xgt diff` makes release-aware taxonomy a tractable step in routine workflows rather than a manual post-processing burden.

Several tools offer complementary functionality worth distinguishing from `xgt`. `TaxonKit` with `gtdb-taxdump` [8] can compute taxonomic changes between GTDB releases by diffing locally downloaded taxonomy dumps. This approach is well suited to bulk offline analysis but requires downloading and maintaining local database files of several gigabytes, and operates on taxon IDs rather than genome

accessions. `xgt diff` operates directly against the live GTDB REST API without any local installation, accepts genome accessions as input, and returns complete taxonomy snapshots at both releases for contextual interpretation. The two approaches are complementary: `TaxonKit` is preferable for large-scale offline batch analysis, while `xgt` is suited to interactive use and integration into API-dependent pipelines. Workflow management systems such as Snakemake [9] and Nextflow [10] can orchestrate `xgt` as a step within larger genomic pipelines, and R packages such as `taxize` provide NCBI-based taxonomy utilities; none of these tools expose GTDB REST API functionality directly, and they are better understood as composable partners than as alternatives to `xgt`.

Current limitations include sequential pagination (throughput bounded by per-page API latency), API-dependent operation (no offline mode), and partial release filtering (the `--release` flag applies only to genome card and metadata endpoints; `search` and `taxon` endpoints always return the current release). Future development will introduce tolerant deserialisation to handle API type changes without tool updates, and parallel pagination with rate-limit management. As GTDB continues to expand, from approximately 317 000 genomes in R207 to over 901 000 in R232 [1], scalable, release-aware programmatic access will become increasingly important. `xgt` is designed to grow with the database and serve as a stable foundation for GTDB-dependent workflows.

Availability of source code and requirements

- Project name: xgt
- Project home page: https://github.com/Ebedthan/xgt
- Operating system(s): Linux (x86_64), macOS (x86_64, Apple Silicon), Windows (x86_64)
- Programming language: Rust (edition 2021, MSRV 1.85)
- Other requirements: None (self-contained binary); Rust 1.85 or later required to build from source
- License: MIT and Apache 2.0 (dual licence)
- RRID: SCR_028473
- bio.tools ID: xgt

Data Availability

The datasets supporting the results of this article are available in the GitHub repository [11].

List of abbreviations

- API: Application Programming Interface;
- CLI: Command-Line Interface;
- CSV: Comma-Separated Values;
- GTDB: Genome Taxonomy Database;
- JSON: JavaScript Object Notation;
- MAG: Metagenome-Assembled Genome;
- REST: Representational State Transfer;
- TLS: Transport Layer Security;

- TSV: Tab-Separated Values.

Consent for publication

Not applicable.

Competing Interests

The authors declare no competing interest.

Funding

Not applicable.

Author's Contributions

Anicet E. T. Ebou: Conceptualization, Methodology, Software, Writing - Original draft. **Dominique K. Koua**: Conceptualization, Methodology, Writing - Review & editing. **Adolphe Zézé**: Supervision, Writing - Review & editing.

Acknowledgements

Not applicable.

References

1. Parks DH, Chuvochina M, Rinke C, Mussig AJ, Chaumeil P-A, Hugenholtz P. GTDB: an ongoing census of bacterial and archaeal diversity through a phylogenetically consistent, rank normalized and complete genome-based taxonomy. *Nucleic Acids Res*. 2022; doi: 10.1093/nar/gkab776.

2. Riesco R, Trujillo ME. Update on the proposed minimal standards for the use of genome data for the taxonomy of prokaryotes. *Int J Syst Evol Microbiol*. Microbiology Society,; 2024; doi: 10.1099/ijsem.0.006300.

3. Clagnan E, Costanzo M, Visca A, Di Gregorio L, Tabacchioni S, Colantoni E, et al.. Culturomics- and metagenomics-based insights into the soil microbiome preservation and application for sustainable agriculture. *Front Microbiol*. Frontiers; 2024; doi: 10.3389/fmicb.2024.1473666.

4. Chaumeil P-A, Mussig AJ, Hugenholtz P, Parks DH. GTDB-Tk: a toolkit to classify genomes with the Genome Taxonomy Database. *Bioinformatics*. 2020; doi: 10.1093/bioinformatics/btz848.

5. Klabnik S, Nichols C. The Rust Programming Language. No Starch Press; ISBN: 978-1-59327-828-1.

6. Chen B, Wu D, Zhang G. GFFx: A Rust-based suite of utilities for ultra-fast genomic feature extraction. *GigaScience*. 2025; doi: 10.1093/gigascience/giaf124.

7. Peter D(2023, March). hyperfine. (Version 1.16.1). https://github.com/sharkdp/hyperfine

8. Shen W, Ren H. TaxonKit: A practical and efficient NCBI taxonomy toolkit. *J Genet Genomics*. 2021; doi: 10.1016/j.jgg.2021.03.006.

9. Mölder F, Jablonski KP, Letcher B, Hall MB, Dyken PC van, Tomkins-Tinch CH, et al.. Sustainable data analysis with Snakemake. F1000Research; doi: 10.12688/f1000research.29032.3.

10. Di Tommaso P, Chatzou M, Floden EW, Barja PP, Palumbo E, Notredame C. Nextflow enables reproducible computational workflows. *Nat Biotechnol*. Nature Publishing Group; 2017; doi: 10.1038/nbt.3820.

11. Ebou A.E.T., Koua D.K., Zézé A. xgt: a command-line interface for the Genome Taxonomy Database with cross-release taxonomic comparison. 2026; https://github.com/Ebedthan/xgt_article

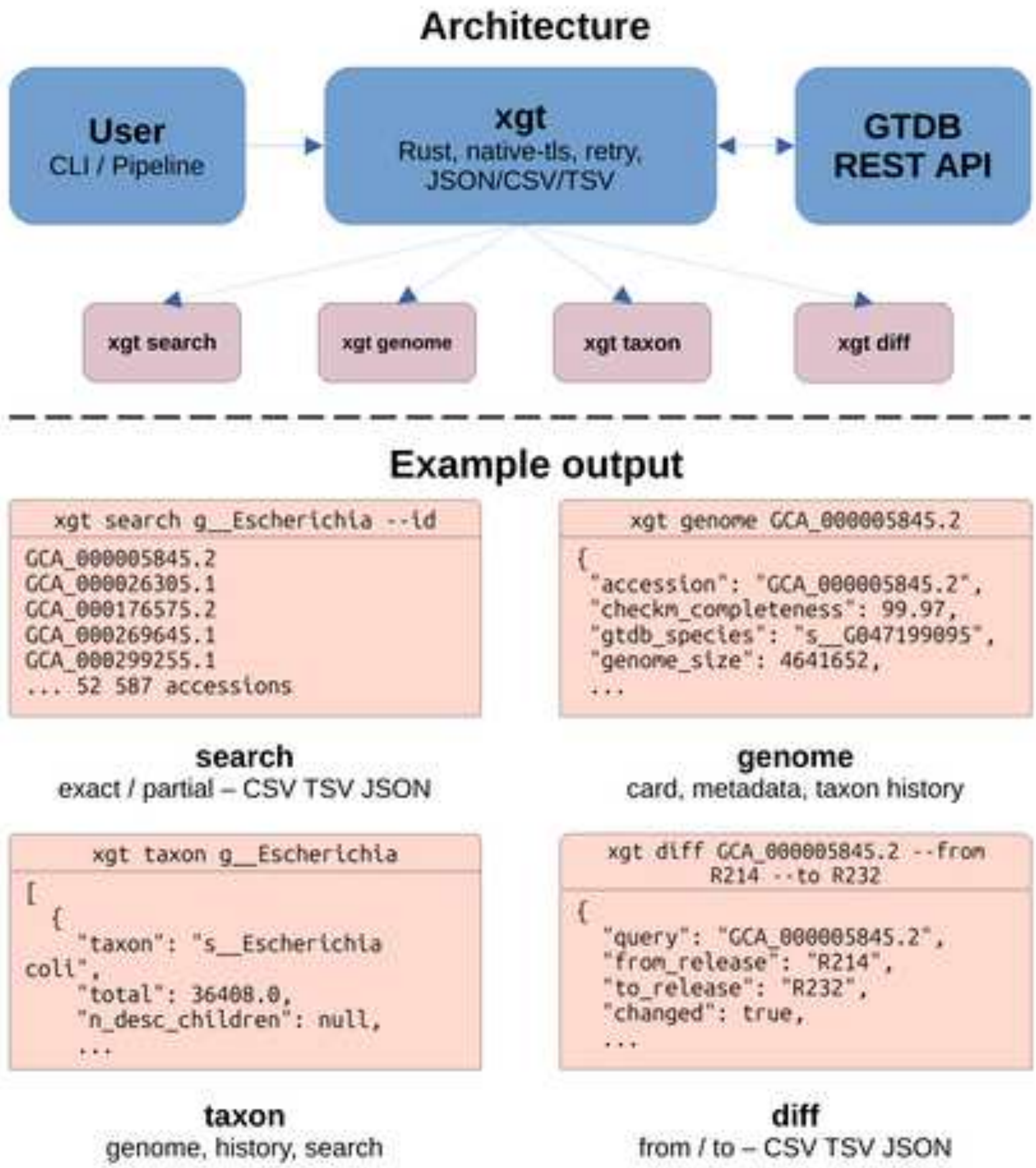
Architecture
User
CLI / Pipeline
xgt
Rust, native-tls, retry,
JSON/CSV/TSV
GTDB
REST API
xgt search
xgt genome
xgt taxon
xgt diff
Example output
xgt search g__Escherichia --id
GCA_000005845.2
GCA_000026305.1
GCA_000176575.2
GCA_000269645.1
GCA_000299255.1
... 52 587 accessions
xgt genome GCA_000005845.2
{
"accession": "GCA_000005845.2",
"checkm_completeness": 99.97,
"gtdb_species": "s__G047199095",
"genome_size": 4641652,
...
search
exact / partial – CSV TSV JSON
genome
card, metadata, taxon history
xgt taxon g__Escherichia
[
{
"taxon": "s__Escherichia coli",
"total": 36408.0,
"n_desc_children": null,
...
xgt diff GCA_000005845.2 --from R214 --to R232
{
"query": "GCA_000005845.2",
"from_release": "R214",
"to_release": "R232",
"changed": true,
...
taxon
genome, history, search
diff
from / to – CSV TSV JSON

Click here to access/download
Supplementary Material
supplementary_R2.pdf

Updated manuscript in docx format with track changes and line numbers

Click here to access/download
Supplementary Material
xgt_article_R2.docx
