# Supplementary material for: xgt: a command-line interface for the Genome Taxonomy Database with cross-release taxonomic comparison
Source: Gigascience. 2026 Aug 14;15:giag086. doi: 10.1093/gigascience/giag086 (PMC13505384; doi:10.1093/gigascience/giag086)
Supplement: Supplementary file 6 [file giag086_reviewer_2_report_original_submission.pdf]

Reviewer Report

Title: xgt: a command-line interface for the Genome Taxonomy Database with cross-release taxonomic comparison

Version: Original Submission **Date:** 7/21/2026

Reviewer name: Reviewer 2

Reviewer Comments to Author:

xgt is an open-source command-line tool written in Rust that wraps the GTDB REST API through four subcommands (search, genome, taxon, and diff). The commands are very well designed from a user's point of view. The completions subcommand is a nice addition, although it is not mentioned in the main text. The main contribution is xgt diff, which reads the taxon-history endpoint and computes per-rank taxonomic changes for a genome between two GTDB releases.I tried to reproduce the small and medium benchmarks on my own macOS machine. I measured xgt at 2.01x the speed of curl+jq for the small query and 3.24x for the medium one, which supports the claim made in the manuscript. I did not run the large benchmark, in order to avoid putting load on the public API.My comments concern two things: (a) given that comparable published tools exist, the claim that the cross-release diff is the first and only tool of its kind may be too strong, and (b) there are several numerical and labelling inconsistencies in the manuscript.Major comments1. TaxonKit is not among the tools compared against (TaxonKit: Shen & Ren, J. Genet. Genomics 2021, https://github.com/shenwei356/gtdb-taxdump#taxid-changes). I would ask you to add the combination of gtdb-taxdump and taxonkit taxid-changelog to Table S3 and to state explicitly how xgt diff differs from them. I expect the difference is that xgt reads the live API, so that no static taxdump is required and an arbitrary pair of releases can be compared on demand. As things stand, the claim that xgt diff is the first and only tool to compute per-rank taxonomic changes between arbitrary GTDB release pairs is not fully supported, although as a 'single' tool it could be only one. I should say that I consider the tool itself genuinely useful, so my suggestion is to soften the novelty claims rather than to drop them.2. As the Current limitations paragraph of the Discussion states, --release applies only to the genome card and metadata endpoints, and search and taxon always return the current release. The command used to assemble the cohort in S1.6, xgt search p__Pseudomonadota --rep --id, does not carry --release either, so what it returns is the R232 representative set as it stood on the date of the analysis (25 May 2026). If that is correct, then the 20,715 genomes in "for all 20 715 species representative genomes assigned to p__Pseudomonadota" are not all of the species representative genomes of the phylum. They would be the subset of the roughly 46,800 R232 representatives that were also present in R214 and for which the diff succeeded. In that case 38.3% is not a rate over all Pseudomonadota representatives, but a rate over the genomes common to the two releases, and it should not be generalised to the representative set as a whole.Minor comments1. S1.5 gives 52,587 accessions for g__Escherichia, but the search panel of Fig. 1 shows "... 36 403 accessions" and the taxon panel shows "total": 36408. The figure may have been made from an older release, or the numbers may be wrong.2. Running the xgt diff command shown in Fig. 1 returns an error. R212 does not appear to be an existing GTDB release. I assume it is a typo for R214, but

please check.3. The JSON in the same xgt diff panel of Fig. 1 has a syntax error. The last line reads "to: "s__G047199095 sp047", where the key should be written "to":.4. The Table S1 caption says "Relative speedup is with respect to the slowest tool per group", but the values appear to be relative to the fastest tool, since xgt is 1.00 in every group.5. Still on Table S1, the speedup for the medium query is given as 4.27 in the main text, which does not match the value in the Relative column. I assume the difference is whether the ratio is taken from the mean or from the median. It would be better to make the text and the table consistent.6. Table S2 is very interesting. The main text discusses only the changes at species and genus level, but the table shows changes of a similar magnitude at family and order level. I think the text should address this.7. Running gtdb_reclassification_analysis.sh (https://github.com/Ebedthan/xgt_article/blob/main/gtdb_reclassification_analysis.sh) with --taxon p__Pseudomonadota --from R214 --to R232 --limit 20 fails with an undefined variable error.

Level of Interest

Please indicate how interesting you found the manuscript: Choose an item.

Quality of Written English

Please indicate the quality of language in the manuscript: Choose an item.

Declaration of Competing Interests

Please complete a declaration of competing interests, considering the following questions:

- Have you in the past five years received reimbursements, fees, funding, or salary from an organisation that may in any way gain or lose financially from the publication of this manuscript, either now or in the future?
- Do you hold any stocks or shares in an organisation that may in any way gain or lose financially from the publication of this manuscript, either now or in the future?
- Do you hold or are you currently applying for any patents relating to the content of the manuscript?
- Have you received reimbursements, fees, funding, or salary from an organization that holds or has applied for patents relating to the content of the manuscript?
- Do you have any other financial competing interests?
- Do you have any non-financial competing interests in relation to this paper?

If you can answer no to all of the above, write 'I declare that I have no competing interests' below. If your reply is yes to any, please give details below.

I declare that I have no competing interests

I agree to the open peer review policy of the journal. I understand that my name will be included on my report to the authors and, if the manuscript is accepted for publication, my named report including any attachments I upload will be posted on the website along with the authors' responses. I agree for my report to be made available under an Open Access Creative Commons CC-BY license (http://creativecommons.org/licenses/by/4.0/). I understand that any comments which I do not wish to be included in my named report can be included as confidential comments to the editors, which will not be published.

I agree to the open peer review policy of the journal

To further support our reviewers, we have joined with Publons, where you can gain additional credit to further highlight your hard work (see: https://publons.com/journal/530/gigascience). On publication of this paper, your review will be automatically added to Publons, you can then choose whether or not to claim your Publons credit. I understand this statement.

Yes Choose an item.
