# Supplementary material for: xgt: a command-line interface for the Genome Taxonomy Database with cross-release taxonomic comparison
Source: Gigascience. 2026 Aug 14;15:giag086. doi: 10.1093/gigascience/giag086 (PMC13505384; doi:10.1093/gigascience/giag086)
Supplement: Supplementary file 7 [file giag086_reviewer_2_report_revision_1.pdf]

Reviewer Report

Title: xgt: a command-line interface for the Genome Taxonomy Database with cross-release taxonomic comparison

Version: Revision 1 **Date:** 8/6/2026

Reviewer name: Reviewer 2

Reviewer Comments to Author:

I thank the authors for the careful revision. Most of the points I raised have been addressed. Four points remain. Two of them follow on from my earlier major comments, where the correction has been made in one place but not in the other.Minor comments1. The novelty claim has been softened in the Discussion, and the new wording there seems right to me. The Results section, however, still reads "To our knowledge, no previously published tool computes per-rank taxonomic changes between arbitrary GTDB release pairs", which is unchanged from the first version. The new Discussion paragraph says that TaxonKit with gtdb-taxdump can compute taxonomic changes between GTDB releases by diffing locally downloaded taxonomy dumps, so the two sections now contradict each other. Please bring the Results sentence into line with the Discussion.2. The Results and Discussion now report the three quantities separately, and the Table S2 caption gives the denominator. That answers my earlier comment. Supplementary Methods S1.6 was not updated, though. It still describes the analysis as covering "species representative genomes assigned to p__Pseudomonadota", gives no counts, and shows the retrieval command with no note that xgt search returns the current release. S1.6 is where a reader will go for the cohort definition, so I would ask you to revise it in the same way as the main text.3. The search panel of Fig. 1 now reads 52 587 accessions, which is what I could get from the current API. The taxon panel of the same figure still reads "total": 36408, so the figure now shows two different numbers for g__Escherichia. It looks as though only the search panel was regenerated. (The response letter gives 52,403 for the regenerated panel, which matches neither the figure nor the API. I assume that is a slip in the letter rather than in the figure.)On the same point, neither number is the size of the genus. xgt search g__Escherichia --field gtdb returns 37,464, and the R232 release files list 37,477 genomes in g__Escherichia. The default search also matches the NCBI taxonomy field, which is where the remaining 15,000 or so records come from. The 36,408 in the taxon panel is the API total for s__Escherichia coli, not for the genus. I would therefore also reword "queries on large genera (g__Escherichia, over 52 000 genomes)" in the Results, since 52 587 is the number of records the query returns rather than the number of genomes in the genus.4. The Discussion states that GTDB grew "from approximately 258 000 genomes in R207 to over 400 000 in R232". The published GTDB statistics give 317,542 genomes for R207 and 901,341 for R232. 258,406 is the R202 figure and 402,709 is R214, so both numbers look to be two releases out. Correcting it strengthens the sentence, since the real growth over that interval is closer to threefold than to the 1.5-fold the present numbers imply.

Level of Interest

Please indicate how interesting you found the manuscript: Choose an item.

Quality of Written English

Please indicate the quality of language in the manuscript: Choose an item.

Declaration of Competing Interests

Please complete a declaration of competing interests, considering the following questions:

- Have you in the past five years received reimbursements, fees, funding, or salary from an organisation that may in any way gain or lose financially from the publication of this manuscript, either now or in the future?
- Do you hold any stocks or shares in an organisation that may in any way gain or lose financially from the publication of this manuscript, either now or in the future?
- Do you hold or are you currently applying for any patents relating to the content of the manuscript?
- Have you received reimbursements, fees, funding, or salary from an organization that holds or has applied for patents relating to the content of the manuscript?
- Do you have any other financial competing interests?
- Do you have any non-financial competing interests in relation to this paper?

If you can answer no to all of the above, write 'I declare that I have no competing interests' below. If your reply is yes to any, please give details below.

I declare that I have no competing interests

I agree to the open peer review policy of the journal. I understand that my name will be included on my report to the authors and, if the manuscript is accepted for publication, my named report including any attachments I upload will be posted on the website along with the authors' responses. I agree for my report to be made available under an Open Access Creative Commons CC-BY license (http://creativecommons.org/licenses/by/4.0/). I understand that any comments which I do not wish to be included in my named report can be included as confidential comments to the editors, which will not be published.

I agree to the open peer review policy of the journal

To further support our reviewers, we have joined with Publons, where you can gain additional credit to further highlight your hard work (see: https://publons.com/journal/530/gigascience). On publication of this paper, your review will be automatically added to Publons, you can then choose whether or not to claim your Publons credit. I understand this statement.

Yes Choose an item.
