# Supplementary material for: xgt: a command-line interface for the Genome Taxonomy Database with cross-release taxonomic comparison
Source: Gigascience. 2026 Aug 14;15:giag086. doi: 10.1093/gigascience/giag086 (PMC13505384; doi:10.1093/gigascience/giag086)
Supplement: Supplementary file 8 [file giag086_supplemental_file.pdf]

Supplementary Material

xgt: a command-line interface for the Genome Taxonomy Database with cross-release taxonomic comparison

Anicet E. T. Ebou   Dominique K. Koua   Adolphe Zézé

Contents

S1 Supplementary Methods **2**
S1.1 Implementation language and design 2
S1.2 Command-line interface and HTTP client 2
S1.3 Data handling and output 2
S1.4 Testing 3
S1.5 Benchmarking protocol 3
S1.6 Taxonomic reclassification analysis 4
S1.7 Representative use case 4

S2 Supplementary Tables **4**

S1 Supplementary Methods

S1.1 Implementation language and design

`xgt` is implemented in Rust (edition 2021, minimum supported version 1.85) [1], a systems programming language that combines memory safety guarantees enforced at compile time with native execution speed and deterministic resource management. The absence of a garbage collector eliminates latency spikes during batch processing, the single-binary compilation model requires no runtime installation, and the strong type system prevents an entire class of null-pointer and buffer-overflow errors without runtime checks. The compiled binary is fully self-contained, requiring no external interpreters, virtual machines, or shared libraries beyond the operating system TLS stack.

The codebase comprises approximately 4 900 lines across eight source files organised into four command modules (`search`, `genome`, `taxon`, `diff`), an API URL-building layer (`api`), a shared utilities layer (`utils`), a CLI argument parser (`cli`), and an entry point (`main`). This modular structure ensures that HTTP logic, output routing, and command-specific behaviour are cleanly separated and independently testable.

S1.2 Command-line interface and HTTP client

The CLI is built with `clap` 4.5, which generates argument parsing, validation, and help text from annotated Rust structs. Shell completion scripts for Bash, Zsh, Fish, and PowerShell are generated at runtime via `clap_complete` 4.5. All subcommands accept input from a positional argument, a file via `--file`, or standard input via `--file -`, enabling direct participation in Unix pipelines.

HTTP communication is handled through `ureq` 3.3 using the `native-tls` backend, which delegates certificate verification to the operating system TLS stack, handling incomplete certificate chains transparently without manual certificate management. Transient errors (HTTP 5xx, 429, and connection failures) are retried up to three times with exponential backoff (1, 2, and 4 seconds, capped at 10 seconds); non-retryable client errors (4xx) fail immediately with messages identifying the offending accession or query. A blocking HTTP client was chosen over an asynchronous architecture to minimise binary size, eliminate runtime dependencies, and simplify error propagation; the throughput cost is most visible for large queries spanning many API pages.

S1.3 Data handling and output

API responses are deserialised into typed Rust structs using `serde` 1.0 and `serde_json` 1.0. Optional fields default to absent when missing from the response, making the tool resilient to additive API schema changes. Field types are declared to match the current API schema, including numeric quality metrics that changed from quoted strings to bare numeric values between GTDB API release cycles; a field whose type changes in a future release would require a corresponding tool update. Mixed-case JSON keys arising from the GTDB API's inconsistent use of camelCase and snake_case are resolved per field, ensuring correct deserialisation regardless of key format.

Search results are paginated automatically: `xgt` fetches all pages sequentially and accumulates results before writing output. The total number of pages is computed as $\lceil \texttt{totalRows}/1000 \rceil$ after the first API response, and all subsequent pages are fetched before any output is produced, eliminating result truncation that affected earlier versions.

All subcommands support `--outfmt csv|tsv|json` and write to standard output by default. The `--out` flag redirects to a single file; `--split` writes one file per query or accession, with shell-safe filenames derived from the query key. An optional `--split-dir` specifies the

output directory. When processing batch inputs, a progress bar is displayed on standard error, preserving stdout for piped downstream tools.

S1.4 Testing

The test suite uses `mockito` 1.7 for HTTP mocking and `tempfile` 3.8 for temporary file handling. Tests cover all HTTP error paths including retry exhaustion and non-retryable 4xx responses; input loading from files and stdin; all output destination variants and filename sanitisation; header-to-value column alignment; taxonomic change computation across zero, one, and all-rank change scenarios; case-insensitive release lookup; pagination accumulation; and JSON round-trip serialisation of diff results. Continuous integration runs `cargo test` and `cargo clippy` via GitHub Actions on Linux (x86_64), macOS (x86_64 and aarch64), and Windows (x86_64) on every push. Code coverage is reported to Codecov via `cargo-llvm-cov`.

S1.5 Benchmarking protocol

Wall-time performance was benchmarked using `hyperfine` 1.20.0 [2], a command-line benchmarking tool that provides mean, median, standard deviation, and minimum elapsed time across repeated runs. Three tools were compared on the `search --id` task, which retrieves all genome accessions for a genus and is the most representative operation for evaluating pagination performance:

- `xgt` 1.0.0 — `xgt search <genus> --id`
- `curl` 8.5.0 + `jq` 1.7 — a manually written shell script issuing sequential `curl` requests and extracting accessions with `jq`
- `python` 3.12 + `requests` 2.31 — a minimal Python script with manual pagination using the `requests` library

Three query sizes were selected to represent the range of genus sizes encountered in practice:

- *small*: *g__Rhizobium* (2 222 accessions, 3 API pages)
- *medium*: *g__Bacillus* (13 875 accessions, 14 API pages)
- *large*: *g__Escherichia* (52 587 accessions, 53 API pages)

Each benchmark was preceded by three warmup runs to warm the TCP connection and server-side response caching, reducing cold-start bias. Ten timed runs were performed per command per genus. Correctness was verified before timing by confirming that all three tools returned identical accession counts for each genus. All tools received identical HTTP parameters (1 000 accessions per page, JSON format).

Hardware and environment: Intel Core i7-8650U (4 cores / 8 threads, 4.2 GHz max), 16 GB RAM, Samsung NVMe SSD, Ubuntu 24.04.4 LTS (kernel 6.17.0-29-generic), residential broadband network.
Date: 21 May 2026. **GTDB release:** R232.

Note on network variability: Because all tools query the same remote API sequentially, wall time is partially determined by network latency and server load. Results from multi-page queries should therefore be interpreted as indicative of real-world workflow efficiency rather than isolated computational performance. For the large query (*g__Escherichia*, 53 pages), coefficients of variation exceeded 100% for all tools, confirming that network jitter dominates at this scale; the median is the more appropriate central tendency measure.

The benchmark script is available at `https://github.com/Ebedthan/xgt_article`.

S1.6 Taxonomic reclassification analysis

To demonstrate the biological utility of xgt diff, we retrieved the 46,827 R232 species representative genomes assigned to p__Pseudomonadota using xgt search. Because the search and taxon endpoints always return data from the current GTDB release (as noted in the tool's limitations), this command returns the representative set as it stood on the date of the analysis (25 May 2026, corresponding to R232). Of these 46,827 accessions, 26,112 (55.8%) were absent from R214 (genomes added to GTDB between releases) and excluded from the diff comparison, leaving 20,715 genomes present in both releases for analysis. Species representatives are the single genome selected by GTDB to anchor each species cluster; restricting the analysis to them provides a conservative estimate of reclassification rates not inflated by redundant or lower-quality assemblies.

Representative accessions were retrieved with:

```
xgt search p__Pseudomonadota --rep --id
```

Taxonomic changes were then computed for each accession with:

```
xgt diff -f accessions.txt --from R214 --to R232 -O json
```

Diff results were written as JSON and parsed with a brace-depth counter to correctly split concatenated pretty-printed JSON objects. For each successfully analysed genome, we recorded whether any rank changed (`changed: true|false`), the number of ranks that changed simultaneously, and the specific old and new taxonomic assignments at each changed rank. Per-rank change frequencies and multi-rank change distributions were computed with a custom Python analysis script available at `https://github.com/Ebedthan/xgt_article`.

Date: 25 May 2026. **GTDB API:** `https://gtdb-api.ecogenomic.org`. **Hardware:** Intel Core i7-8650U (16 GB RAM, Ubuntu 24.04.4 LTS). **Tool:** `xgt` 1.0.0.

S1.7 Representative use case

The following four-step workflow illustrates how `xgt` integrates into a metagenomics study to assess taxonomic stability across GTDB releases. A researcher who has recovered metagenome-assembled genomes (MAGs) assigned with GTDB R214 taxonomy can determine which assignments remain stable in R232:

```
# Step 1: retrieve representative accessions for the focal genus
xgt search g__Rhizobium --rep --id > rhizobium_reps.txt

# Step 2: fetch full genome cards for all representatives
xgt genome -f rhizobium_reps.txt -O csv -o rhizobium_cards.csv

# Step 3: compute per-rank taxonomic changes between releases
xgt diff -f rhizobium_reps.txt --from R214 --to R232 \
-O csv -o rhizobium_diff.csv

# Step 4: identify genomes with species-level reclassifications
awk -F',' '$5 == "species" && $4 == "true"' rhizobium_diff.csv
```

This workflow, which would previously require custom API scripting, manual JSON parsing, and separate pagination handling, executes entirely within `xgt` and produces analysis-ready CSV files. The `--split` flag can be used to write one file per genome for downstream per-genome pipelines.

S2 Supplementary Tables

Table S1: Wall-time benchmarks for the `search --id` task across three genus sizes. Values are mean, median, and standard deviation over 10 runs (3 warmup runs; GTDB release R232, residential broadband, Intel Core i7-8650U). For *g__Escherichia* the coefficient of variation exceeds 100% due to network jitter over 53 API pages; the median is the more appropriate central tendency measure. Relative speedup is with respect to xgt (the fastest tool in each group).

Genus	Tool	Mean (s)	Median (s)	SD (s)	Min (s)	Relative
g__Rhizobium	`xgt`	0.408	0.392	0.058	0.338	1.00
	`python+requests`	0.705	0.709	0.068	0.629	1.80
	`curl+jq`	1.193	1.204	0.076	1.026	3.07
g__Bacillus	`xgt`	1.520	1.502	0.137	1.322	1.00
	`python+requests`	2.771	2.784	0.108	2.628	1.85
	`curl+jq`	7.014	6.412	1.125	6.268	4.26
g__Escherichia	`xgt`	14.753	5.287	30.465	4.647	1.00
	`python+requests`	20.994	13.097	21.432	9.678	2.47
	`curl+jq`	36.707	25.201	36.491	24.634	4.76

Table S2: Taxonomic reclassification rates between GTDB releases R214 and R232 for species representative genomes assigned to *p__Pseudomonadota* ($n = 20\,715$, genomes present in both R214 and R232 out of 46 827 R232 representatives). Percentages in the last column are of all 20,715 analysed genomes.

Rank	Genomes reclassified	% of total
domain	0	0.0
phylum	3	0.0
class	4	0.0
order	2,139	10.3
family	2,927	14.1
genus	3,231	15.6
species	4,077	19.7
Any rank	7,941	38.3

Table S3: Comparison of `xgt` with related tools for GTDB data access. `curl/wget` are general-purpose HTTP tools that can query the API but provide no pagination, retry logic, structured parsing, or typed output. GTDB-Tk classifies novel genome assemblies against the GTDB reference phylogeny and does not query the REST API [3]. TaxonKit with `gtdb-taxdump` [4] can compute cross-release taxonomic changes from locally downloaded GTDB taxonomy dumps, but requires maintaining local database files and operates on taxon IDs rather than genome accessions. Many users also interact with GTDB programmatically via custom Python scripts or Jupyter notebooks; while these approaches offer flexibility, they require per-user implementation of pagination, error handling, and output formatting that `xgt` provides out of the box. ✓: supported; ×: not supported; N/A: not applicable to the tool's primary function.

Feature	`xgt`	`curl/wget`	GTDB-Tk	TaxonKit+dump
GTDB API queries	✓	✓	×	×
Batch input (file/stdin)	✓	×	✓	✓
Automatic pagination	✓	×	N/A	N/A
Retry with backoff	✓	×	N/A	N/A
JSON / CSV / TSV output	✓	JSON only	✓	TSV only
Cross-release diff	✓	×	×	✓
No local database needed	✓	✓	×	×
Accession-based input	✓	✓	✓	×
No runtime dependencies	✓	✓	×	×
Shell completions	✓	×	×	×

References

[1] Steve Klabnik and Carol Nichols. *The Rust Programming Language*. No Starch Press, 2018. ISBN 978-1-59327-828-1.

[2] David Peter. hyperfine: A command-line benchmarking tool, 2023. URL `https://github.com/sharkdp/hyperfine`.

[3] Pierre-Alain Chaumeil, Aaron J Mussig, Philip Hugenholtz, and Donovan H Parks. GTDB-Tk: A toolkit to classify genomes with the Genome Taxonomy Database. *Bioinformatics*, 36(6):1925–1927, March 2020. ISSN 1367-4803. doi: 10.1093/bioinformatics/btz848.

[4] Wei Shen and Hong Ren. TaxonKit: A practical and efficient NCBI taxonomy toolkit. *Journal of Genetics and Genomics*, 48(9):844–850, September 2021. ISSN 1673-8527. doi: 10.1016/j.jgg.2021.03.006.
